# Systematic benchmark of substructure search in molecular graphs - From Ullmann to VF2

**DOI:** 10.1186/1758-2946-4-13

**Published:** 2012-07-31

**Authors:** Hans-Christian Ehrlich, Matthias Rarey

**Affiliations:** 1 Center for Bioinformatics, University of Hamburg, Bundestraße 43, 20146 Hamburg, Germany

**Keywords:** Substructure search, Subgraph isomorphism, Algorithm, Benchmark, SMARTS, Chemical pattern search

## Abstract

**Background:**

Searching for substructures in molecules belongs to the most elementary tasks in cheminformatics and is nowadays part of virtually every cheminformatics software. The underlying algorithms, used over several decades, are designed for the application to general graphs. Applied on molecular graphs, little effort has been spend on characterizing their performance. Therefore, it is not clear how current substructure search algorithms behave on such special graphs. One of the main reasons why such an evaluation was not performed in the past was the absence of appropriate data sets.

**Results:**

In this paper, we present a systematic evaluation of Ullmann’s and the VF2 subgraph isomorphism algorithms on molecular data. The benchmark set consists of a collection of 1235 SMARTS substructure expressions and selected molecules from the ZINC database. The benchmark evaluates substructures search times for complete database scans as well as individual substructure-molecule pairs. In detail, we focus on the influence of substructure formulation and size, the impact of molecule size, and the ability of both algorithms to be used on multiple cores.

**Conclusions:**

The results show a clear superiority of the VF2 algorithm in all test scenarios. In general, both algorithms solve most instances in less than one millisecond, which we consider to be acceptable. Still, in direct comparison, the VF2 is most often several folds faster than Ullmann’s algorithm. Additionally, Ullmann’s algorithm shows a surprising number of run time outliers.

## Background

Today’s drug discovery faces a constantly growing number of commercially available or synthetically accessible compounds maintained in large databases [1,2]. In order to efficiently search such databases, computational search strategies comprising various search criteria have been developed over more than four decades [3-14]. Search criteria range from retrieving the one exact compound over selecting compounds via substructure features to the application of various similarity measures. In the following, we focus on methods that test compounds for the presence of certain functional groups or substructures.

Modeling molecular structures as labeled graphs has a long tradition and gives the basis for modern cheminformatics methods. A graph-based representation is chemically intuitive and forms a solid theoretical foundation for computer-aided processing. Furthermore, graphs allow the substructure search problem to be solved by graph isomorphism techniques, i.e., searching molecules for substructures is equivalent to testing two labeled graphs for subgraph isomorphism. The subgraph isomorphism problem is well studied [15-17] and one of the oldest and most applied algorithms [18-22] was introduced by Ullmann in 1976 [7]. Over the years that followed, only a few subgraph isomorphism methods were introduced [11,16,23], the most recent being the VF2 algorithm [12].

Until now, each comparison of (sub-)graph isomorphism algorithms [16,17] only employs synthetic graph data. The data is most often constructed to show the algorithms’ behavior on medium to large graphs. Therefore, it is unclear how these algorithms behave on rather small graphs like molecular data. To our knowledge, no subgraph isomorphism comparison directly addresses the problem of searching chemical substructures in molecules. One of the main reasons why such a benchmark was not performed in the past was the lack of suitable and publicly available benchmark data sets.

This article describes such various data sets and discusses the differences between the Ullmann and the VF2 subgraph isomorphism algorithm applied on substructures and molecules. In the following, we introduce the graph theoretical concepts, summarize the two algorithms of interest, introduce different benchmark data sets and compare the algorithms’ performance in various molecular modeling scenarios.

## Preliminaries

For almost 150 years, chemists have used chemical and structural formulas to represent molecules. A structural formula is closely related to the mathematical concepts of graphs which makes graph theory and algorithms directly applicable in cheminformatics.

### Graph theoretical background

A *graphG*=(*V*,*E*) is defined by a set of nodes *V * and a set of connecting edges *E*. The edges of an *undirected* graph have no fixed orientation and if labels are assigned to nodes or edges the graph is denoted as *labeled*. If a path from each node to every other nodes exists, the graph is called *connected*. In the following, all graphs are labeled, undirected and connected except when stated otherwise.

#### Subgraph isomorphism

Two graphs *G*_1_=(*V*_1_,*E*_1_) and *G*_2_=(*V*_2_,*E*_2_) are *isomorphic* if a bijective projection between nodes *V *_1_ and nodes *V *_2_ exists such that two nodes from *V *_1_ are connected by an edge from *E*_1_ if and only if their image nodes in *V *_2_ are connected by an edge from *E*_2_. An *induced subgraph* of a graph *G*=(*V*,*E*) is defined as a graph *G*^*′*^=(*V*^*′*^,*E*^*′*^) whose nodes *V*^*′*^ are a subset of *V * and whose edges *E*^*′*^ are all possible edges from *E* that connect two nodes in *V*^*′*^. An *induced subgraph isomorphism* between a query graph *G*_1_ and a target graph *G*_2_ exists if *G*_1_ is isomorphic to an induced subgraph of *G*_2_, i.e., the query graph *G*_1_ is a subgraph of the target graph *G*_2_.

The problem of finding an isomorphic induced subgraph is believed to be a problem for which no efficient solution exists, i.e., it belongs to the class of NP-complete problems [5,24]. Therefore, every subgraph isomorphism algorithm will show exponential run times with respect to the input graph size.

#### Molecular graphs

A *molecular graph* is given by nodes and edges that represent atoms and bonds, respectively. Often nodes and edges are labeled with atom and bond properties. Obviously, molecular graphs are undirected. The number of edges connecting each node is limited by the number of covalent bonds an atom can form. Therefore, the number of edges in a molecular graph linearly depends on the number of nodes.

Molecules are equal or isomorphic if their molecular graphs are isomorphic and the labels of the atoms and bonds are equal to the labels of their mapped atoms and bonds respectively. When two molecules differ in size, one can be a substructure of the other, i.e., a subgraph isomorphism between the two molecules exists. The small number of atoms and the linear atom degree allow for a fast subgraph isomorphism test on molecules.

#### Substructure graphs

**Figure 1:**
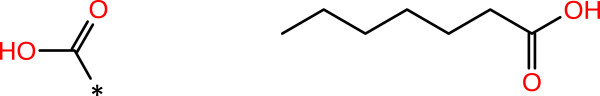
**Carboxylic acid pattern and heptonic acid.** A carboxylic acid pattern (left) with ‘*’ indicating any atom. Heptonic acid (right).

A *substructure graph* can be a molecule fragment, e.g., a functional group, or a more generalized construct. For example, a single halogen node might represent a fluorine, chlorine, bromine or iodine atom. The same applies to edges, e.g., an edge is either a single or a double bond. In the following, we will use substructure graphs with such general labels. Figure 1 shows an example.

Substructure graphs are compared with molecules to detect subgraph isomorphisms. The goal is to determine the presence or location of a functional group or a specific molecular structure. Nodes and edges are mapped to atoms and bonds in accordance with their labels. Since edges are explicitly assigned to bonds, the detected isomorphic subgraph might not be induced, i.e., non-circular substructures can be mapped to circular molecule parts.

For a clear differentiation, we will use the terms atoms and bonds for molecular target graphs and nodes and edges for query substructure graphs.

### Algorithm 1

## Substructure pattern languages

A substructure graph can be formulated by using a substructure pattern language like SMILES Arbitrary Target Specification (SMARTS) [25], Sybyl Line Notation (SLN) [26] or Wiswesser Line Notation (WLN) [27]. All languages define a substructure graph in a textual line notation similar to a molecule’s chemical formula. They allow the definition of a substructure’s topology and node and bond properties, including logical alternatives. SMARTS even provides the opportunity to specify additional information like a chemical environment. In this study, all substructures are formulated as SMARTS expressions.

## Methods

The Ullmann and the VF2 algorithms are two algorithms that solve the subgraph isomorphism problem. Applied to substructure and molecular graphs, they can be used to detect substructures in molecules. Both algorithms calculate an exact solution, i.e., the exact substructure must be present, and their application is not restricted to a special class of graphs, i.e., is not limited to molecular graphs.

### Ullmann algorithm

**Figure 2:**
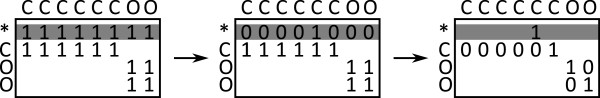
**Iteration of Ullmann algorithm.** One step of the Ullmann algorithm. The initial compatibility matrix (left) shows carboxylic acid substructure nodes as rows and heptonic acid molecule atoms as columns. A non-zero entry indicates the compatibility of a node-atom pair. Zero entries are not shown. In the current row, indicated in gray, the algorithm choses one compatible node-atom mapping (middle) and refines all unprocessed rows (right). The algorithm continues with the next row. Figure 3 illustrates the refinement.

The Ullmann algorithm [7] is a backtracking procedure that employs a relaxation-based refinement step to reduce the search space. It operates on a *n*×*m*matrix *M* of boolean values, where *n* is the number of substructure nodes and *m* the number of molecule atoms. An entry at position (*ij*) marks the compatibility of labels for substructure node *i* and molecule atom *j*. Additionally, it uses a boolean vector *f * of length *m* marking mapped atoms. Algorithms Algorithm 1 and Algorithm 2 show Ullmann’s match and refinement procedure. Figure 2 illustrates one step of the algorithm. 

**Figure 3:**
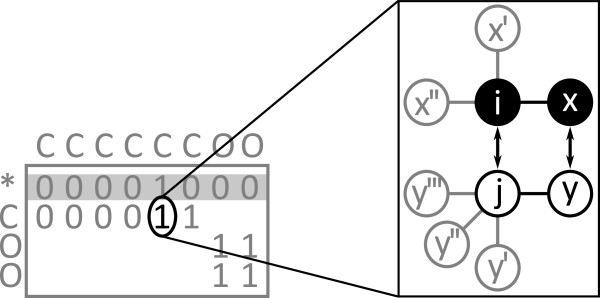
**Refinement of Ullmann algorithm.** Ullmann refinement step. For a mapping of node *i* to atom *j*, all adjacent nodes *x* must have at least one valid mapping *y*. If this condition is not fulfilled, the mapping (*i*,*j*) is invalid and the corresponding matrix entry at (*i*,*j*) is set to zero.

### Algorithm 2

The refinement is the crucial step of the algorithm. It evaluates the surrounding of every possible node-atom mapping. For a valid mapping, every neighbor node must have a compatible atom as illustrated in Figure 3. Otherwise, the mapping is invalid which is marked by setting the corresponding matrix entry to zero. The evaluation takes place for every possible mapping downstream the current row and is repeated until all remaining mappings are valid.

Although the refinement procedure is the key for an efficient reduction of the search space it does not take full advantage of topological constraints. For example, in the case of a small substructure and a large molecule, it evaluates entries topologically too far away from already mapped node-atom pairs.

## VF2 Algorithm

**Figure 4:**
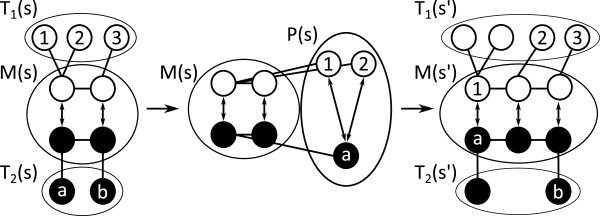
**Iteration of VF2 algorithm.** One VF2 iteration. The algorithm extends the current solution *M(s)* of state *s* by one candidate *(1,a)* chosen from *P(s)*. *T*_1_(*s*) and *T*_2_(*s*) show the nodes adjacent to mapped atoms and nodes.

The VF2 algorithm [12] iteratively extends a partial solution using a set of feasibility criteria to decide whether to extend or backtrack. It operates on an intermediate algorithm state *s* which is composed of a partial solution *M(s)* and adjacency sets *T*_1_(*s*) and *T*_2_(*s*). A pair (*nm*)∈*M*(*s*) represents an atom-node mapping of the partial solution. *M*_1_(*s*) and *M*_2_(*s*) describe the atoms and nodes, respectively, that belong to the partial solution. *T*_1_(*s*) and *T*_2_(*s*) hold atoms and nodes adjacent to atoms in *M*_1_(*s*) and nodes in *M*_2_(2), respectively. The algorithm modifies the state *s* in two steps. From the sets *T*_1_(*s*) and *T*_2_(*s*), it creates a candidate set *P(s)* of atom-node pairs with compatible labels. Then, it explores every candidate (*nm*)∈*P*(*s*) that fulfills the feasibility rules *F*_*syn*_or backtracks if *P(s)* is empty. Figure 4 graphically depicts one step of the algorithm. 

**Figure 5:**
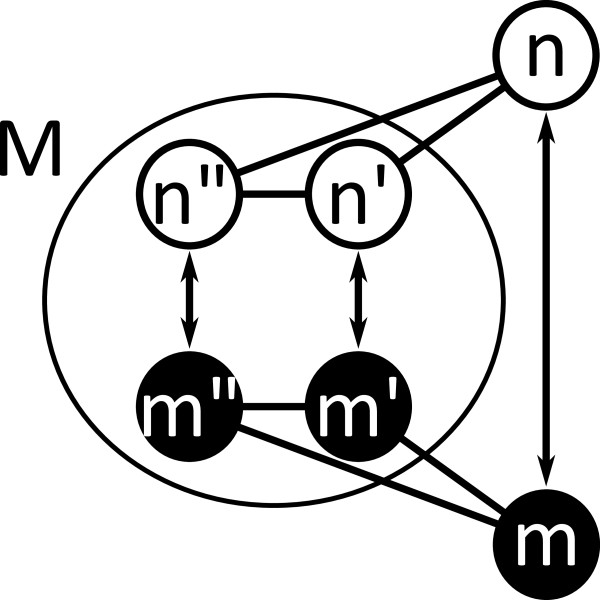
**VF2 feasibility rule for node cardinality.** VF2 feasibility rule for node cardinality. The rule guaranties a one-to-one mapping of edges in the current solution *M*(*s*). For a candidate mapping (*n*,*m*), all atoms (*n*^*′ *^and *n*^*′′ *^in *M*_1 _(*s*)) adjacent to *n* must be mapped to the corresponding nodes (*m*^*′ *^and *m" *in *M*_1 _(*s*)) adjacent to *m*. Otherwise the candidate mapping is not feasible.

**Figure 6:**
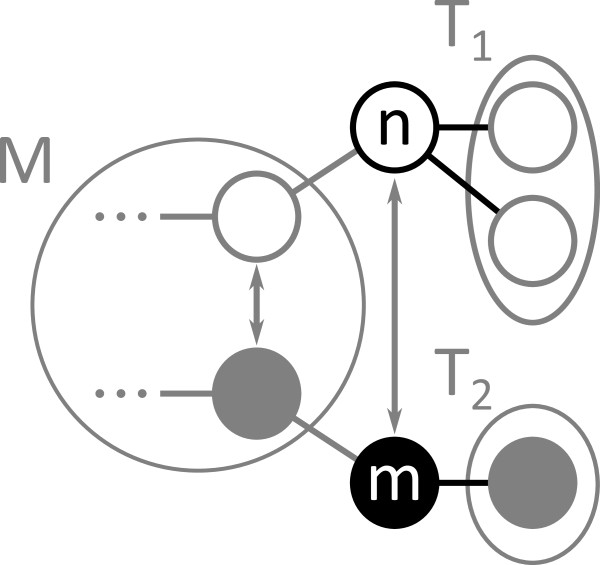
**VF2 feasibility rule for node cardinality (1-look-ahead).** VF2 feasibility rule for node cardinality (1-look-ahead). The rule prohibits an extension of the current solution *M*(*s*) by candidates with a substructure cardinality that can not be fully mapped onto the graph. In the given example, node *m* has one edge into *T*_2_(*s*) and is mapped to atom *n* with a cardinality of two. Therefore, the mapping is feasible.

*F*_*syn*_(*s*,*n*,*m*) (Equation 1) describes the feasibility of candidates *(n,m)* in state *s*. It is composed out of two terms, *R*_*adj*_ (Equation 2) and *R*_*inout*_ (Equation 3). The first feasibility rule *R*_*adj*_ guarantees that each atom *n*^*′*^ and node *m*^*′*^ adjacent (*Adj*) to the atom *n* and node *m* of a candidate pair (*n*,*m*) are mapped to each other in the partial solution (*n*^*′*^,*m*^*′*^)∈*M*(*s*). The second rule *R*_*inout*_performs a 1-look-ahead in the search procedure based on the nodes’ cardinality (*Card*) and allows an early pruning of the search tree. Figure 5 and Figure 6 give an illustration of the feasibility rules.

(1) $$\begin{array}{cc} F_{\text{syn}}(s,n,m) & =R_{\text{adj}}\wedge R_{\text{inout}} \end{array}$$

(2) $$\begin{array}{cc} R_{\text{adj}} & =(\forall n^{\prime }\in M_{1}(s)\cap \text{adj}(G_{1},n))\exists m^{\prime }\in \text{adj}(G_{2},m)|(n^{\prime },m^{\prime })\in M(s))\;\\ & \;\wedge (\forall m^{\prime }\in M_{2}(s)\cap \text{adj}(G_{2},m))\exists n^{\prime }\in \text{adj}(G_{1},n)|(n^{\prime },m^{\prime })\in M(s)) \end{array}$$

(3) $$\begin{array}{cc} R_{\text{inout}} & =\text{Card}(\text{adj}(G_{1},n)\cap T_{1}(s))\;\geq \;\text{Card}(\text{adj}(G_{2},m)\cap T_{2}(s)) \end{array}$$

The problem of reaching the same state, i.e., the same partial solution *M(s)*, via different paths is handled by imposing an arbitrary total order ≺ onto the subgraph nodes and processing only smallest feasible candidates with regard to that order. Therefore, feasible candidates (*n*_*i*_,*m*_*j*_) in *P(s)* are not processed if *m*_*k*_≺*m*_*j*_∈*P*(*s*).

The main difference between the two algorithms is the way they account for the topology of the substructure. The Ullmann algorithm processes a compatibility matrix top-down. In every step it fixes one node-atom mapping and checks all other possible assignments for validity. Therefore, it processes substructure nodes in an non-topological, arbitrary order. In contrast, the VF2 iteratively adds node-atom pairs to a current solution and therefore directly explores the substructure’s topology.

## Algorithm 3

## Substructure pattern formulation for efficient computation

The formulation of substructure patterns is a tedious task. Most pattern languages are difficult to read and even more difficult to write, especially when defining isomeric or tautomeric structures. As a result, substructure formulations are focused on a correct chemical representation of a pattern. That formulation might be suboptimal for computational processing. Therefore, we present simple guidelines to optimize patterns for the search in molecules.

For an optimal formulation, the substructure must be in an order that allows an early processing of unusual nodes and edges, rare fragments and functional groups. Obviously, certain elements are more common than others. The same applies for substructure nodes that define a high number of atom properties or are part of an aromatic system. Unusual edges define aromatic bonds or those with a high bond order. Therefore, we write optimized substructures such that nodes with the rarest element, highest property specification and aromaticity as well as high order or aromatic bond definitions occur first. Additionally, we place substructure parts that are rather common or difficult to process at the end of the formulation. Nodes that specify generic atoms, hydrogen atoms, carbon atoms, and ring atoms are common. Chemical environments are difficult to process for most search algorithms, since they enforce an additional search step.

In the following we perform every pattern reformulation by hand. Nevertheless, both algorithms are well suited for an automated optimization process. Ullmann’s algorithm processes substructure nodes according to their row numbers in the compatibility matrix. Since row numbers are assigned arbitrarily, they can resemble the order employed by applying the given optimization rules. The VF2 uses an arbitrary node relation to obtain a total order. Therefore, the optimized order can be directly used.

## Data sets

Both algorithms are tested in different application setups like complete database scans, substructure-based filter scenarios and individual substructure-molecule searches. The tests show the dependency of the algorithm run times on substructure formulation, substructure size and molecule size.

The data sets comprise 1336 SMARTS from the literature [28-37] and molecules out of ZINC lead-like and ZINC everything database [1]. All data sets are provided in Additional file 1.

### Substructure search set

Molecule size is a crucial factor with respect to the algorithmic search time. To explore the influence of molecule size, we select a subset from the initial 1336 SMARTS. All duplicate expressions, expressions with errors, extensions and those that define isotopes or are disconnected are removed. The resulting set comprises 1235 SMARTS whose property overview is given in the Additional file 2: Table S1. SMARTS allows the explicit formulation of hydrogen atoms and the definition of atom environments. When explicit hydrogen atoms are used a search procedure must evaluate all hydrogen atoms, which roughly doubles the number of atoms to be evaluated. Atom environments induce an additional search step during the actual search procedure. In order to circumvent misinterpretations of the results, we group the SMARTS patterns by the presence/absence of explicit hydrogens and recursive environments into individual sets. The Additional file 2: Table S2 – S19 give detailed statistics on SMARTS properties for every set.

The final sets contain all SMARTS patterns for which 100 molecules containing the pattern could be randomly selected from ZINC lead-like and ZINC everything. Table 1 shows the number of SMARTS for which the selection process was successful. The molecular property distribution of each set is similar to the corresponding ZINC database as shown in the Additional file 2: Table S23 – S24.

**Table 1:** SMARTS, ZINC lead-like, ZINC everything test sets

	**all SMARTS**		**ZINC lead-like set**		**ZINC everything set**	
	**no H nodes**	**H nodes**	**no H nodes**	**H nodes**	**no H nodes**	**H nodes**
no recursion	504	432	347	56	400	43
recursion	234	65	48	18	106	39

All processable SMARTS split by the presence/absence of explicit hydrogen nodes and recursive environment specifications and the subsets used in the ZINC lead-like and ZINC everything set.

### Molecule search set

Substructure size is the second major factor regarding pattern matching time. A set to measure its impact is composed by randomly selecting molecules from ZINC lead-like containing all-in-all 80 different substructures of various size. The presence of so many substructures in a single molecule is rather rare but selecting molecules with less patterns gives poor results. A selection was only possible for the set of SMARTS having no explicit hydrogen nodes and no recursive environments. The other three sets contain patterns of much higher complexity which are rarely present in one molecule or patterns that are designed to be complementary to each other, e.g., PAINS.

### PAINS substructure set

For a detailed case study, we choose 16 PanAssayINterferenceStructures(PAINS) described by Baell et al. [38] as ‘filter family A’. The PAINS substructures should describe unspecific binders in protein-protein interaction assays. PAINS were originally given in SLN and converted to SMARTS by Rajarshi Guha using Cactvs [39]. The converted PAINS patterns include hydrogen atoms and recursive environments. The PAINS’s property distribution is shown in the Additional file 2: Table S20 and Additional file 3: Figure S2 – S5 depict each substructure.

### Worst-case test

Since highly symmetric molecules impose a challenge for substructure search algorithms, we test a phenylring query against a fulleren target as a worst-case search scenario.

### Database subset

The database subset comprises the first 100.000 molecules from ZINC lead-like as of February 12th, 2011 and is designed to resemble a complete database. Its property distribution is similar to that of the full ZINC lead-like database as shown in Additional file 2: Table S25.

## Results and discussion

Search speed is measured on a single Intel(R) Xeon(R) CPU E5630 2.53GHz core. Each matching is repeated 400 times and average values are recorded. Average matching times are raw matching times excluding File I/O, molecule initialization and post-processing of search results, i.e., matching time only.

We are aware of the fact that the evaluation is done with an example implementation of both algorithms that most likely has some room for optimization. Nevertheless, we believe that our results allow general conclusions regarding the algorithms’ behavior on molecular data.

### Overall search speed

**Figure 7:**
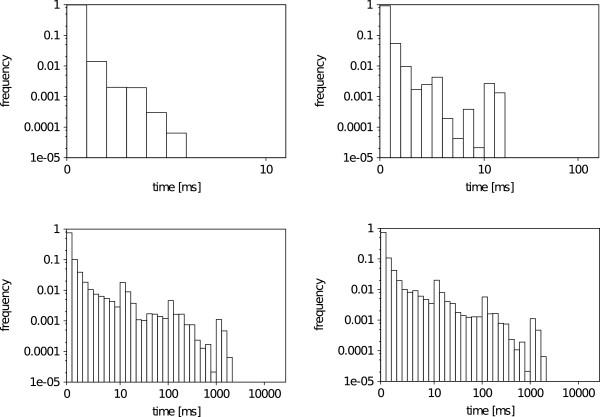
**Overall run time histogram.** Histogram over VF2 (top) and Ullmann (bottom) matching times on the Substructure Search Set. The algorithms search for the first (left) and all (right) occurrence(s) of the substructure. All plots are double logarithmic and times are given in milliseconds (ms).

An overview of the VF2 and Ullmann matching times is shown in Figure 7. The times are measured on the 46900 substructure-molecule pairs of the Substructure Search Set. Both substructure algorithms search for all occurrences of each substructure. The histograms show that both algorithms have most match times in a range below 1 milliseconds (ms) (92.3% VF2, 73,4% Ullmann) with a median of 0.04ms for the VF2 and 0.1ms for the Ullmann, respectively. While the maximum VF2 matching time is below 30ms, the Ullmann shows times of more than 100ms for 1.12% (5352 pairs) and more than 1000ms for 0.22% (104 pairs) of the data set. Interestingly, the Ullmann search times do not change drastically in the case where the search is constrained to the first occurrence of each substructure. In contrast, the VF2 outlier times drop down by one half. In conclusion, both algorithms can solve most instances in reasonable time and the median run times differ by a factor of 2.5 betwenn VF2 and Ullmann’s algorithm. In rare cases, the Ullmann algorithm is up to 1000 times slower than the VF2.

### Explicit vs. implicit hydrogens

**Figure 8:**
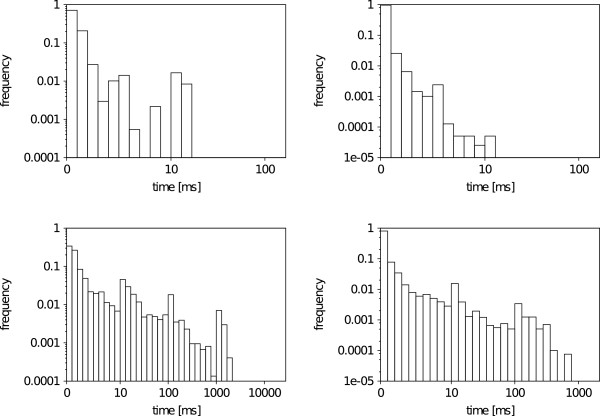
**Explicit vs. Implicit hydrogens run time histogram.** Histogram of VF2 (up) and Ullmann (down) matching times with (left) and without (right) explicit hydrogens on the Substructure Search Set. The algorithms search for all occurrences of the substructure. All plots are double logarithmic and times are given in milliseconds (ms).

A closer analysis of Ullmann and VF2 matching times reveals a slight increase in run times for SMARTS patterns with explicit hydrogens, which is documented by the histograms in Figure 8. The median search times of the VF2 are 0.08ms for substructures with only implicit hydrogens and 0.19ms with explicit hydrogens, 0.22ms and 1.09ms for the Ullmann, respectively. In accordance, the maximum run time of the VF2 doubles, while that of the Ullmann algorithm is roughly four times larger. The reason for an increase in run times is twofold. About 50% of atoms in a small molecule are hydrogens. Therefore, when matching patterns with explicit hydrogens, in contrast to patterns with only implicit hydrogens, all hydrogen atoms have to be evaluated. This doubles the number of evaluated atoms during the search, and hence, increases the run time. Additionally, for every hydrogen node, an explicit placement must be found, as opposed to the comparison of the sheer number of hydrogens attached to an atom. This raises the number of evaluated atoms as well as the number of found mappings, and therefore increases the run time.

### Recursion vs. no recursion

**Figure 9:**
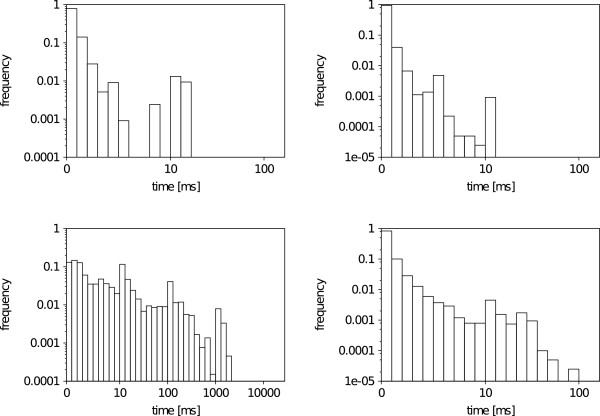
**Recursion vs. no Recursion run time histogram.** Histogram of VF2 (up) and Ullmann (down) matching times with (left) and without (right) recursive environments on the Substructure Search Set. The algorithms search for all occurrences of the substructure. All plots are double logarithmic and times are given in milliseconds (ms).

An interesting aspect of the SMARTS pattern language is the ability to recursively define the chemical environment of an atom. To match a pattern that includes one or more nodes with atom environments, a subgraph search algorithm has to recursively perform a subgraph isomorphism test during the actual search. Figure 9 shows the impact on matching times when recursive environments are defined. Median run times for the VF2 are 0.04 ms for SMARTS without and 0.35 ms for SMARTS with environment specifications, 0.15 ms and 4.87 ms for Ullmann’s algorithm, respectively. Surprisingly, the Ullman algorithm is much more sensitive to recursive patterns. The presence of environment specifications can lead to a 30 times increase in Ullmann matching times while VF2 times maximal rise by a factor of two. The sensitivity is due to the fact that Ullmann’s algorithm creates a matrix that represents all possible mappings of nodes to atoms. Since most recursive environments are rather small, the construction and evaluation of such a matrix represents a computational overhead that is reflected in an increase of the overall search time.

### Molecule size

**Figure 10:**
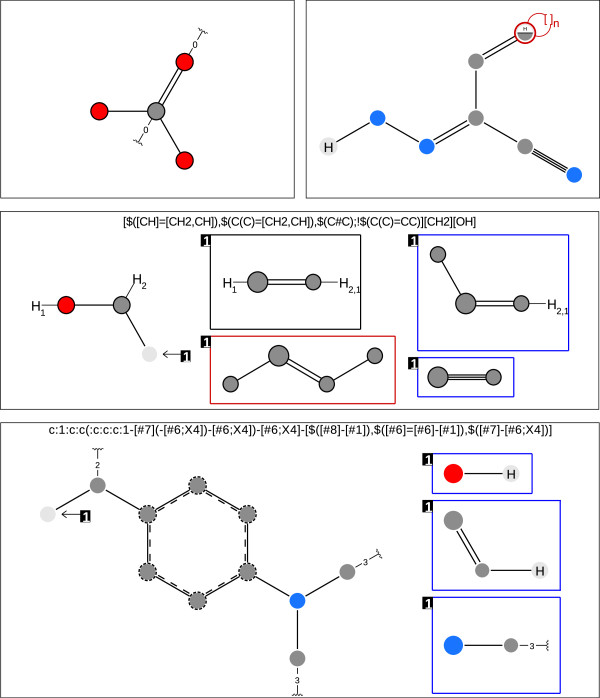
**Molecule size experiment patterns.** Depiction of SMARTS pattern with no explicit hydrogens and no recursion (top-left), explicit hydrogens and no recursion (top-right), no explicit hydrogens and recursion (middle) and with explicit hydrogens and recursive atom environments (bottom). The legend can be found in the Additional file 3: Figure S1. Depictions are created by SMARTSViewer [42].

**Figure 11:**
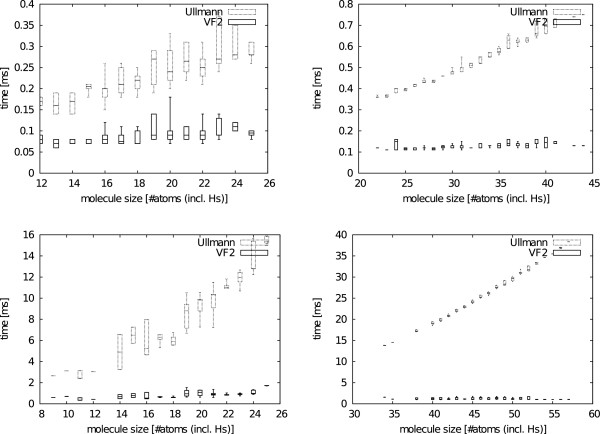
**Molecule size search example.** Run time comparison between Ullman and VF2 searching for all substructure occurrences with various molecule sizes. The different plots show a linear increase in run time with respect to the molecule size. The top-left pattern does not include explicit hydrogens nor recursive environments. The top-right pattern does include explicit hydrogens but not recursive environments. The bottom-left pattern does not include explicit hydrogens but recursive environments. The bottom-right pattern includes explicit hydrogens and recursive environments. Figure 10 shows a graphical depiction of all four patterns. Times are given in milliseconds (ms).

In order to explore the influence of molecule size we examine 469 substructure-molecule pairs from the Substructure Search Set. As almost all results are similar, we chose only some representative substructure-molecule pairs shown in Figure 10. All figures, given in Additional file 1, show a significantly smaller matching time for the VF2 and a linear influence of the molecule size on the matching time. The difference between VF2 and Ullmann matching times becomes even more prominent when examining the cases where explicit hydrogens (Figure 11 top-left), recursive environments (Figure 11 bottom-right) or both (Figure 11 bottom-left) are present. The linear impact of the molecule size on the run time is explained by the constant number of bonds an atom can form as can be obtained from a theoretical analysis of backtracking algorithms for subgraph isomorphism [40,41]. 

### Subgraph size

**Figure 12:**
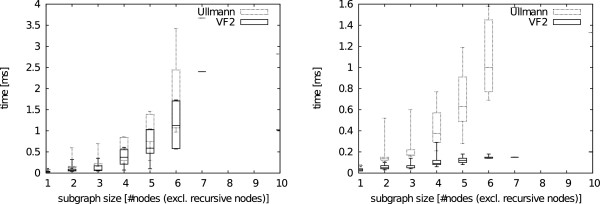
**Subgraph size search example.** Run time comparison between Ullman and VF2 searching for all (left) and the first (right) substructure occurrence(s) with varying subgraph size. The plots show an exponential increase in run time with respect to the substructure size. Times are given in milliseconds (ms).

The impact of subgraph size regarding the matching time was evaluated with a meaningful test set for substructures with only implicit hydrogens and no recursive environments. Unfortunately, a suitable test set could only be constructed for SMARTS patterns without explicit hydrogens and recursive environments. From observing 100 molecules in which at least 80 substructures with different size could be matched, we assume an exponential run time development with increasing subgraph size for both algorithms. The exponential increase seems to be slower for the VF2 in all cases. An example is given in Figure 12 and all plots are provided in Additional file 1. The difference in matching times drastically decreases when only the presence of a substructure, rather than all occurrences, is of interests. The exponential match time of both algorithms regarding the substructure size is again in agreement with a theoretical analysis of the subgraph isomorphism problem [40,41]. 

### Worse-case test

As a worse-case substructure search scenario, we test a phenyl-ring query against a C70 fullerene target. The Ullmann finds the first occurrence in 51.11 ms and all matches in 106.94 ms. The VF2 is about 130 times faster when it solves the problem for the first occurrence (0.39 ms) and about 5 times when searching for all matches (21.67ms). Clearly, the phenyl-fullerene example is not the worse-case when considering SMARTS substructures. Substructures with explicit hydrogen nodes or recursive atom environments yield much higher run times. Nevertheless, the phenyl-fullerene experiment gives good guidance on how the Ullmann and VF2 algorithms behave on highly symmetrical structures.

### Complete database search

Often substructure search algorithms are used in database search scenarios in which a database is scanned for all molecules that contain a given query structure. Even though most database search systems are able to eliminate a large number of molecules from the actual subgraph isomorphism search using screening techniques [10,22,41,43-46], a remarkable number of molecules might remain. The following test simulates a sequential subgraph isomorphism test over a large set of molecules. We search all 1235 patterns from the Substructure Search Set against the Database Subset and measure the complete time to identify all molecules which contain such a substructure. Since the majority of the first 100.000 molecules of the ZINC lead-like database do not contain a given pattern, the search time is dominated by the algorithm’s ability to quickly identify the non-occurrence of a substructure in a molecule. A good screening method would of course enrich the molecules submitted to the isomorphism test with molecules containing the substructure of interests. Nevertheless, testing both algorithms for the ability of quickly detecting molecules without a given pattern will reveal further insights into the algorithmic behavior. This test is only performed once, as minor changes in run time do not affect the order of magnitude.

**Figure 13:**
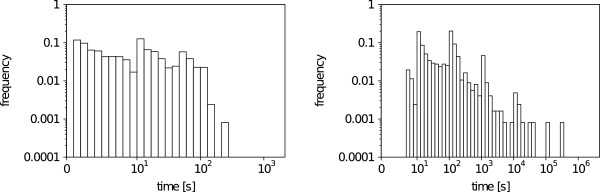
**Database scan run time histogram.** Run time histogram for the VF2 (left) and Ullmann (right) when searching the first 100.000 molecules from ZINC lead-like for the first substructure occurrences. Both plots are double logarithmic and times are given in seconds(s).

From the two histograms in Figure 13, it is clear that the VF2 algorithm is much faster in sequentially scanning a large number of molecules. The median search time of the VF2 is 2.84 s and 38.7 s for the Ullmann. The VF2 algorithm finishes 53.06% of the search queries below 10s and 97.61% below 10^2^ s, while the Ullmann completes 3.73% below 10s, 54.24% below 10^2^ s, 92.36% below 10^3^ s (16.6 min), 98.76% below 10^4^ s (2.78 h) and 99.85% below 10^5^s (27.78 h). All in all, in rare instances a database search system that uses the Ullmann algorithm might need over a day to give results for a single query, even though, most of the molecules might be eliminated from the subgraph isomorphism test.

### Parallelization scaling

The subgraph isomorphism problem is nearly perfectly suited for parallel computing when matching one query structure against many target structures. One simple but effective solution is a parallelization by data separation of the target structures. An alternative is an algorithm level parallelization based on the algorithms’ recursion. Since most substructure searches are below 1ms and most molecules consist of less than 100 atoms, a parallelization of one substructure against one target search is most likely not as efficient as searching in parallel on the data level. The situation might change when searching large query substructures against large target structures, e.g., searching for motifs in proteins.

In order to evaluate the efficiency of data level parallelization, we test both algorithms with the same data separation strategy on the PAINS Substructure Set against the complete ZINC lead-like database on different numbers of CPU cores. The target structures are split into equal blocks such that each core gets the query structures and a the same number of molecules. The measurement on one core is performed in sequential and parallel mode so that the computational overhead for parallelization becomes directly present. Detailed tables on the matching times and scaling factors on different numbers of cores can be found in Additional file 2: Table S26 – S27.

Both algorithm show good scaling behavior on all instances. On 8 cores the search times are decreased by an average factor of 5.6 for the VF2, and 6.92 for Ullmann’s algorithm respectively. The overall slightly better scaling of the Ullmann algorithm can be explained by the longer matching times. Longer matching times reduce the parallelization overhead relative to the matching time.

### SMARTS pattern case studies

To explore the possibility of reducing search speed by rearranging the subgraph formulation we created three different formulations for each substructure of the PAINS Substructure Set. The *original* substructure formulation as created by Cactvs, an *optimized* version by applying the re-formulation guidelines described in the “Substructure Pattern Formulation” section, and an *anti-optimized* version by applying the rules in reverse. All three formulations are searched against the complete ZINC lead-like database.

As can be observed from the two most extreme cases shown in Table 2, the VF2 algorithm shows run time decreases of up to 13.37 times for the optimized substructure formulations. In accordance, the run time increases up to 15.64 times for the anti-optimized formulation. Surprisingly, the Ullmann algorithm shows no significant change in run time, neither for the optimized nor for the anti-optimized version in all test cases.

**Table 2:** Optimization run time examples

	**Ull. time [s]**	**Ull. speedup**	**VF2 time [s]**	**VF2 speedup**	**matches**
**PAINS 4**					
original	157139.71	1.00	170.42	1.00	11699
optimized	157027.63	1.00	168.56	1.01	11699
anti-opt.	154195.33	1.02	2664.49	-15.64	11699
PAINS 12 original	3119.04	1.00	1698.42	1.00	9056
optimized	2142.41	1.46	142.28	11.94	9056
anti-opt.	3077.34	1.01	1675.40	1.01	9056

Two examples of searching the PAINS Substructure Set against the complete ZINC lead-like database. Ullmann and VF2 times in seconds and speed ups are shown. Results for all 16 PAINS are given in the Additional file 2: Table S28 – S29 and the re-formulated PAINS in Additional file 2: Table S30.

### Ullman faster than VF2

In almost all test-cases, we see a superior matching performance of VF2 compared to Ullmann’s algorithm. In order to exclude the possibility of errors in our time measurements, we re-calculate the benchmarks for all cases in which Ullmann’s algorithm shows a smaller matching time than the VF2. The number of repetitions for each search call is increased to 100.000 to increase the time measurement accuracy. Table 3 shows the re-measurement for 10 examples. Clearly, the first measurements were sufficiently accurate and in all these cases the Ullmann outperformed the VF2. To investigated if the subgraph formulation might be unfortunate for the VF2 algorithm, the test is repeated with optimized substructure formulations. The matching times given in Table 3 show that the VF2 is faster in all cases when given an optimized substructure formulation.

**Table 3:** Ullmann faster than VF2 without optimization examples

**SMARTS**	**Ullmann time**	**VF2 time**
	**[ms]**	**[ms]**
[#6]C(=[O,SX2])[CX4]C(=[O,SX2])[#6]	0.868	0.948
[O,SX2]=C([#6])[CX4]C(=[O,SX2])[#6]	0.654	0.271
[#6]C(=[O,SX2])C(=[O,SX2])[#6]	0.938	1.046
[O,SX2]=C([#6])C(=[O,SX2])[#6]	0.668	0.203
[a]˜*˜*-[CH3]	0.479	0.601
[CH3]-*˜*˜[a]	0.209	0.074
[C](=O)([C,c,O,S])[C,c,O,S]	0.400	0.558
O=[C]([C,c,O,S])[C,c,O,S]	0.403	0.144
[CD3H0,R](=[SD1H0])([ND2H1,R])([ND2H1,R])	0.251	0.510
[SD1H0]=[CD3H0,R]([ND2H1,R])([ND2H1,R])	0.242	0.076
[nD3H0,R](˜[OD1H0])(a)a	0.290	0.435
[OD1H0]˜[nD3H0,R](a)a	0.290	0.091
[R](-*(-*))˜*˜*˜*˜[a]	2.082	2.774
[a]˜*˜*˜*˜[R](-*(-*))	1.764	0.906
c([OH])c([OH])c([OH])	0.581	0.708
[OH]cc([OH])c([OH])	0.581	0.274
c1([OH])c(O[CH3])cccc1	0.805	0.947
[OH]c1c(O[CH3])cccc1	0.797	0.169
c1([OH])ccc(O[CH3])cc1	0.74	0.922
[OH]c1ccc(O[CH3])cc1.	0.734	0.193

Examples for SMARTS without explicit hydrogens and recursive environments for which the Ullmann algorithm shows a superior run time compared to the VF2. Time measurements are averages over 100.000 search repetitions in milliseconds. Times are shown for the original SMARTS formulation (top) and an optimized version (bottom) according to our guidelines.

## Conclusions

We presented, to our knowledge, the first comparison between Ullmann and VF2 subgraph isomorphism algorithm on molecular data and the first data set to perform such a benchmark. Using SMARTS as molecular substructure language, we explored the influence of substructure and molecular size as well as the usage of explicit hydrogen nodes and recursive environment specification on the matching time. Both algorithms where additionally tested for the use in complete database scans and their ability for data-based parallelization. Additionally, we presented an optimization strategy to reduce matching times by substructure pattern reformulation.

In conclusion, the VF2 algorithm outperforms the Ullman in all test cases when supplied with a favorable substructure formulation and seems to be more robust in terms of run time outliers. Even though the VF2 is generally faster, both algorithms perform most single substructure-molecule searches in times below one millisecond, which seems acceptable for most cheminformatics applications. Nevertheless, we recommend using the VF2 algorithm for molecular substructure searching in cheminformatics software because it shows a general run time superiority of about one order of magnitude.

The syntactic formulation of a substructure in terms of arrangement might be a critical point for the underlying subgraph isomorphism algorithm. Our experiments show that the VF2 algorithm is sensitive to the substructure’s formulation while the Ullmann algorithm is not. Therefore, other subgraph isomorphism algorithms might show the same sensitivity and need to be experimentally tested.

Fortunately, the subgraph reformulation rules as shown here have not to be done by hand. The VF2 algorithm is based on a precalculated node order which can be manipulated following the reformulation rules. Due to the sensitivity of the VF2 algorithm for node rearrangements, the algorithm has further room for optimization.

## Competing interests

The authors declare that they have no competing interests.

## Authors’ contributions

H-CE implemented the presented software components, collected the data and performed the comparison studies. MR supervised the project. Both authors read and approved the final manuscript.

## Supplementary Material

Additional file 1 **Additional data (Additional file**1**). /datasets/smarts/literature_Hs_noRec.smarts. SMARTS substructures with hydrogens and no recursion.**
SMARTS substructure patterns with hydrogens and no recursive atom environments.
/datasets/smarts/literature_Hs_rec.smarts. SMARTS substructures with hydrogens and recursion.
SMARTS substructure patterns with hydrogens and with recursive atom environments.
/datasets/smarts/literature_noHs_noRec.smarts. SMARTS substructures without hydrogens and no recursion.
SMARTS substructure patterns without hydrogens and no recursive atom environments.
/datasets/smarts/literature_noHs_rec.smarts. SMARTS substructures without hydrogens and with recursion.
SMARTS substructure patterns without hydrogens and with recursive atom environments.
/datasets/smarts/pains_p_m150_antioptimized.txt. PAINS substructures anti-optimized.
PAINS substructures as SMARTS in anti-optimized formulation.
/datasets/smarts/pains_p_m150_antioptimized.txt. PAINS substructures anti-optimized.
PAINS substructures as SMARTS in anti-optimized formulation.
/datasets/smarts/pains_p_m150_original.txt. PAINS substructures original.
PAINS substructures as SMARTS in original formulation as obtained from the literature.
/datasets/substructure_search_set/literature_Hs_noRec.smarts.everything.benchmarkset. Substructure Search Set, explicit hydrogens and no recursion, ZINC everything
Search set to test the run time influence of the molecule size. Substructures are in SMARTS and do contain explicit hydrogens but no recursive atom environments. For each substructure pattern 100 molecules that contain the pattern were selected at random from ZINC everything. Substructures and molecules are given as space separated SMARTS and SMILES.
/datasets/substructure_search_set/literature_Hs_rec.smarts.everything.benchmarkset. Substructure Search Set, explicit hydrogens and with recursion, ZINC everything.
Search set to test the run time influence of the molecule size. Substructures are in SMARTS and do contain explicit hydrogens and recursive atom environments. For each substructure pattern 100 molecules that contain the pattern were selected at random from ZINC everything. Substructures and molecules are given as space separated SMARTS and SMILES.
/datasets/substructure_search_set/literature_noHs_noRec.smarts.everything.benchmarkset. Substructure Search Set, no explicit hydrogens and no recursion, ZINC everything.
Search set to test the run time influence of the molecule size. Substructures are in SMARTS and do not contain explicit hydrogens or recursive atom environments. For each substructure pattern 100 molecules that contain the pattern were selected at random from ZINC everything. Substructures and molecules are given as space separated SMARTS and SMILES.
/datasets/substructure_search_set/literature_noHs_rec.smarts.everything.benchmarkset. Substructure Search Set, no explicit hydrogens and with recursion, ZINC everything.
Search set to test the run time influence of the molecule size. Substructures are in SMARTS and do not contain explicit hydrogens but recursive atom environments. For each substructure pattern 100 molecules that contain the pattern were selected at random from ZINC everything. Substructures and molecules are given as space separated SMARTS and SMILES.
/datasets/substructure_search_set/literature_Hs_noRec.smarts.lead-like.benchmarkset. Substructure Search Set, explicit hydrogens and no recursion, ZINC lead-like.
Search set to test the run time influence of the molecule size. Substructures are in SMARTS and do contain explicit hydrogens but no recursive atom environments. For each substructure pattern 100 molecules that contain the pattern were selected at random from ZINC lead-like. Substructures and molecules are given as space separated SMARTS and SMILES.
/datasets/substructure_search_set/literature_Hs_rec.smarts.lead-like.benchmarkset. Substructure Search Set, explicit hydrogens and with recursion, ZINC lead-like.
Search set to test the run time influence of the molecule size. Substructures are in SMARTS and do contain explicit hydrogens and recursive atom environments. For each substructure pattern 100 molecules that contain the pattern were selected at random from ZINC lead-like. Substructures and molecules are given as space separated SMARTS and SMILES.
/datasets/substructure_search_set/literature_noHs_noRec.smarts.lead-like.benchmarkset. Substructure Search Set, no explicit hydrogens and no recursion, ZINC lead-like.
Search set to test the run time influence of the molecule size. Substructures are in SMARTS and do not contain explicit hydrogens or recursive atom environments. For each substructure pattern 100 molecules that contain the pattern were selected at random from ZINC lead-like. Substructures and molecules are given as space separated SMARTS and SMILES.
/datasets/substructure_search_set/literature_noHs_rec.smarts.lead-like.benchmarkset. Substructure Search Set, no explicit hydrogens and with recursion, ZINC lead-like.
Search set to test the run time influence of the molecule size. Substructures are in SMARTS and do not contain explicit hydrogens but recursive atom environments. For each substructure pattern 100 molecules that contain the pattern were selected at random from ZINC lead-like. Substructures and molecules are given as space separated SMARTS and SMILES.
/datasets/substructure_search_set/literature_noHs_rec.smarts.lead-like.benchmarkset. Substructure Search Set, no explicit hydrogens and with recursion, ZINC lead-like.
Search set to test the run time influence of the molecule size. Substructures are in SMARTS and do not contain explicit hydrogens but recursive atom environments. For each substructure pattern 100 molecules that contain the pattern were selected at random from ZINC lead-like. Substructures and molecules are given as space separated SMARTS and SMILES.
/datasets/molecule_search_set/literature_noHs_noRec.smarts.everything.80.benchmarkset. Molecule Search Set ZINC everything.
Search set to test the run time influence of the substructure size. Substructures are in SMARTS and do not include explicit hydrogen nodes or recursive atom environments. For each molecule 80 substructures that are contained in the molecule were selected at random from ZINC everything. Molecules and substructures are given as space separated SMILES and SMARTS.
/datasets/worst_case.benchmarkset. Worst Case Set.
A worst-case substructure search scenario of searching for a phenyl-ring in a highly symmetrical fullerene. Substructure and molecule are in SMARTS and SMILES.**/datasets/zinc_lead-like_2011-02-12_first100k.smi. First 100k Molecules of ZINC lead-like.**
The first 100.000 molecules of the ZINC lead-like database. Molecules are in SMILES.
/results/molecule/allPlots.lead-like.all.eps. Molecule Search Experiment ZINC lead-like.
Experiment to test the run time influence of the substructure size. Plots are box plots showing subgraph size vs. run time for Ullmann and VF2. Both algorithms are set to find all occurrences of a substructure. Molecules were chosen at random from ZINC lead-like.
/results/molecule/allPlots.lead-like.first.eps. Molecule Search Experiment ZINC lead-like.
Experiment to test the run time influence of the substructure size. Plots are box plots showing subgraph size vs. run time for Ullmann and VF2. Both algorithms are set to find first occurrences of a substructure. Molecules were chosen at random from ZINC lead-like.
/results/subgraph/allPlots.lead-like.all.eps. Subgraph Search Experiment ZINC lead-like.
Experiment to test the run time influence of the molecule size. Plots are box plots showing molecule size vs. run time for Ullmann and VF2. Both algorithms are set to find all occurrences of a substructure. Molecules were chosen at random from ZINC lead-like.
/results/subgraph/allPlots.lead-like.first.eps. Subgraph Search Experiment ZINC lead-like.
Experiment to test the run time influence of the molecule size. Plots are box plots showing molecule size vs. run time for Ullmann and VF2. Both algorithms are set to find first occurrences of a substructure. Molecules were chosen at random from ZINC lead-like. Click here for file

Additional file 2 **Supplementary Information (Additional file**2**). Table S1.** Profile over the number of property occurrences of all 1235 SMARTS sub-structures.
**Table S2.** Profile over the number of property occurrences of 738 SMARTS substructures without explicit hydrogens.
**Table S3.** Profile over the number of property occurrences of 497 SMARTS substructures with explicit hydrogens.
**Table S4.** Profile over the number of property occurrences of 936 SMARTS substructures without recursive atom environments.
**Table S5.** Profile over the number of property occurrences of 299 SMARTS substructures with recursive atom environments.
**Table S6.** Profile over the number of property occurrences of 504 SMARTS substructures without hydrogen atoms and without recursion.
**Table S7.** Profile over the number of property occurrences of 234 SMARTS substructures without hydrogen atoms and with recursion.
**Table S8.** Profile over the number of property occurrences of 432 SMARTS substructures with hydrogen atoms and without recursion.
**Table S9.** Profile over the number of property occurrences of 65 SMARTS substructures with hydrogen atom and with recursion.
**Table S10.** Profile over the number of property occurrences of 469 SMARTS substructures used in ZINC lead-like benchmark set.
**Table S11.** Profile over the number of property occurrences of 347 SMARTS substructures with no hydrogen atoms and no recursion in ZINC lead-like benchmark set.
**Table S12.** Profile over the number of property occurrences of 48 SMARTS substructures with no hydrogen atoms and recursion in ZINC lead-like benchmark set.
**Table S13.** Profile over the number of property occurrences of 56 SMARTS substructures with hydrogen atoms and no recursion in ZINC lead-like benchmark set.
**Table S14.** Profile over the number of property occurrences of 18 SMARTS substructures with hydrogen atoms and recursion in ZINC lead-like benchmark set.
**Table S15.** Profile over the number of property occurrences of 588 SMARTS substructures used in ZINC everything benchmark set.
**Table S16.** Profile over the number of property occurrences of 400 SMARTS substructures with no hydrogen atoms and no recursion in ZINC everything benchmark set.
**Table S17.** Profile over the number of property occurrences of 106 SMARTS substructures with no hydrogen atoms and recursion in ZINC everything benchmark set.
**Table S18.** Profile over the number of property occurrences of 43 SMARTS substructures with hydrogen atoms and no recursion in ZINC everything benchmark set.
**Table S19.** Profile over the number of property occurrences of 39 SMARTS substructures with hydrogen atoms and recursion in ZINC everything benchmark set.
**Table S20.** Profile over the number of property occurrences of 16 PAINS patterns.
**Table S21.** Profile for all 2516375 from ZINC lead-like.
**Table S22.** Profile for all 14059666 form ZINC everything.
**Table S23.** Profile 61500 molecules selected from ZINC lead-like for the substructure search set.
**Table S24.** Profile 76800 molecules selected from ZINC everything for substructure search set.
**Table S25.** Profile first 100000 molecules selected from ZINC lead-like.
**Table S26.** Ullmann search times in seconds for PAINS substructures as SMARTS in optimized formulation against the complete ZINC lead-like. Scaling factors (SFs) represent the speed up in comparison to the sequential time.
**Table S27.** VF2 search times in seconds for PAINS substructures as SMARTS in optimized formulation against the complete ZINC lead-like. Scaling factors (SFs) represent the speed up in comparison to the sequential time.
**Table S28.** Ullmann match times in seconds of the PAINS Substructure Set against the complete ZINC lead-like database. All 16 PAINS are given in the original, an optimized, and an anti-optimized substructure formulation in the SI.
**Table S29.** VF2 match times in seconds of the PAINS Substructure Set against the complete ZINC lead-like database. All 16 PAINS are given in the original, an optimized, and an anti-optimized substructure formulation in Table 30.
**Table S30.** SMARTS expressions used in optimization experiment given as taken from literature (original), optimized by the given rule set (optimized) and anti-optimized applying the rule set in reverse (anti-optimized). Click here for file

Additional file 3 **Supplementary Information (Additional file**3**). Figure S1.** Depiction of SMARTS pattern with no explicit hydrogens and no recursion (top-left), explicit hydrogens and no recursion (top-right), no explicit hydrogens and recursion (bottom-left), and with explicit hydrogens and recursive atom environments (bottom-right). Depictions are created by SMARTSViewer [42].
**Figure S2.** Visual dipiction of PAINS patterns 1-4 created with SMARTSViewer [42].
**Figure S3.** Visual dipiction of PAINS patterns 5-8 created with SMARTSViewer [42].
**Figure S4.** Visual dipiction of PAINS patterns 9-12 created with SMARTSViewer [42].
**Figure S5.** Visual dipiction of PAINS patterns 13-16 created with SMARTSViewer [42]. Click here for file
