# Supplementary material for: Systematic benchmark of substructure search in molecular graphs - From Ullmann to VF2
Source: J Cheminform. 2012 Jul 31;4:13. doi: 10.1186/1758-2946-4-13 (PMC3586954; doi:10.1186/1758-2946-4-13)
Supplement: Additional file 1 — Additional data (Additional file1). /datasets/smarts/literature_Hs_noRec.smarts. SMARTS substructures with hydrogens and no recursion. SMARTS substructure patterns with hydrogens and no recursive atom environments. /datasets/smarts/literature_Hs_rec.smarts. SMARTS substructures with hydrogens and recursion. SMARTS substructure patterns with hydrogens and with recursive atom environments. /datasets/smarts/literature_noHs_noRec.smarts. SMARTS substructures without hydrogens and no recursion. SMARTS substructure patterns without hydrogens and no recursive atom environments. /datasets/smarts/literature_noHs_rec.smarts. SMARTS substructures without hydrogens and with recursion. SMARTS substructure patterns without hydrogens and with recursive atom environments. /datasets/smarts/pains_p_m150_antioptimized.txt. PAINS substructures anti-optimized. PAINS substructures as SMARTS in anti-optimized formulation. /datasets/smarts/pains_p_m150_antioptimized.txt. PAINS substructures anti-optimized. PAINS substructures as SMARTS in anti-optimized formulation. /datasets/smarts/pains_p_m150_original.txt. PAINS substructures original. PAINS substructures as SMARTS in original formulation as obtained from the literature. /datasets/substructure_search_set/literature_Hs_noRec.smarts.everything.benchmarkset. Substructure Search Set, explicit hydrogens and no recursion, ZINC everything Search set to test the run time influence of the molecule size. Substructures are in SMARTS and do contain explicit hydrogens but no recursive atom environments. For each substructure pattern 100 molecules that contain the pattern were selected at random from ZINC everything. Substructures and molecules are given as space separated SMARTS and SMILES. /datasets/substructure_search_set/literature_Hs_rec.smarts.everything.benchmarkset. Substructure Search Set, explicit hydrogens and with recursion, ZINC everything. Search set to test the run time influence of the molecule size. Substructures are in SMARTS and do [file 1758-2946-4-13-S1.pdf]

Supplementary Information

Not processable SMARTS:

set without explicit hydrogens and no recursive environment:
[CH3,CQ1H2,CQ1H1] (Q is special character)
[OQ1X1,OQ2X2,NQ3X3,NQ2X2,NQ1X1]
[CQ1X4]~[CQ2X4]~[OQ1X2]
[CQ1X4]~[CQ3X3](~[OQ1X1])~[OQ1X2]
[NQ1X3]~[CQ3X3](~[OQ1X1])~[NQ1X3]
[NQ1X3]~[CQ2X4]~[CQ3X3](~[OQ1X1])~[OQ1X2]
S.S.S (disconnected)
[CD3H0]([ND1H2])([ND1H2])([ND2H1]).[CD3H0]([ND1H2])([ND1H2])([ND2H1])
[#16X2H0][!#16].[#16X2H0][!#16]
[F,Cl,Br,I].[F,Cl,Br,I].[F,Cl,Br,I]
[cR1]1[cR1][cR1][cR1][cR1][cR1]1.[cR1]1[cR1][cR1][cR1][cR1][cR1]1
[#16X2H0][!#16].[#16X2H0][!#16]
([Cl]).([c])
([!-0!-1!-2!-3!-4].[!+0!+1!+2!+3!+4])
[C,S,P](=O)[OH].[C,S,P](=O)[OH].[C,S,P](=O)[OH].[C,S,P](=O)[OH]
[OH]c1cc([OH])cc2=[O+]C(=C([OH])Cc21)c3cc([OH])c([OH]) (unclosed ring 3)
[nH1]ncoc1=O (unclosed ring 1)

set without explicit hydrogens and with recursive environments:
[$([NH2]-c),NQ1H3,NQ2H2,NQ3H1,NQ2H1,$(Cl-),$(Br-),$(I-)] (Q is special character)
[CH3,cH3,CQ1H2,cQ1H2,CQ1H1,cQ1H1]~*~[$([NH2]-A),$([OH]-*);!+;!-]
[CH3,cH3,CQ1H2,cQ1H2,CQ1H1,cQ1H1]~*(~[OQ1X1,oQ1X1,OQ2-
X2,oQ2X2,NQ3X3,nQ3X3,NQ2X2,nQ2X2,NQ1X1,nQ1X1])~[$([NH2]-A),$([OH]-*);!+;!-]
[$([NH2]-A),$([OH]-*);!+;!-]~*(~[OQ1X1,oQ1X1,OQ2X2,oQ2X2,NQ3X3,nQ3X3,NQ2X2,nQ2X2,NQ1X1,nQ1X1;!+;!-])~[$([NH2]-
A),$([OH]-*);!+;!-]
(~[OQ1X1,oQ1X1,OQ2X2,oQ2X2,NQ3X3,nQ3X3,NQ2X2,nQ2X2,NQ1X1,nQ1X1])(~[$([NH2]-A),$([OH]-);!+;!-])~*~[$([NH2]-
A),$([OH]-*);!+;!-]
a1a[$([NH2]-a),NQ1H3,NQ2H2,NQ3H1,NQ2H1,nQ1H3,nQ2H2,nQ3H1,
a12aaaaa1aa[$([NH2]-a),NQ1H3,NQ2H2,NQ3H1,NQ2H1,nQ1H3,nQ2H2,nQ3H1,nQ2H1,$(Cl-*),$(Br-*),$(I-*);!+]2
[OH;!$([OH][C,S,P])=O)] (unmatched bracket)
[C;!D4;!D1;!R;$(C(=O));$(C([n])[!N;!D1][!D1])]-;!@[n;!$(n@[#6](=O));!$((C=O)(n)([#7]))]
[NX3;H2,H1;!$(NC=O)].[NX3;H2,H1;!$(NC=O)] (disconnected)
[$([NX3](=O)=O),$([NX3+](=O)[O-])][!#8].[$([NX3](=O)=O),$([NX3+](=O)[O-])][!#8]
[$([*R2]([*R])([*R])([*R]))].[$([*R2]([*R])([*R])([*R]))]
[$([NX3H2,NX4H3+]),$([NX3H](C)(C))][CX4H]([(unexpected end)
[$([$(*-[NX2-]-[NX2+]#[NX1]),$(*-[NX2]=[NX2+]=[NX1-])]),$([$([NX1-]=[NX2+]=[NX1-]),$(
([Cl!$(Cl~c)].[c!$(c~Cl)])
[NX3;H2,H1;!$(NC=O)].[NX3;H2,H1;!$(NC=O)]
[$([$([NX4]=O),$([NX4+][O-,#0])])] (includes #0)
[$(C=C),$(C#C),a][CH2,CH][O,S][$([CH]=O),$([CH]=S),$(C(C)=O),$(C(C)=S),[a]] ('[]' inside '[]')
[#6][O,SX2,N][O,SX2,N][$([CH]=O),$([CH]=S),$(C(C)=O),$(C(C)=S),[a]]
[$(NH2)!:c],$([NH1]([CX4])!:c),$([NH0]([CX4])([CX4])!:c)] (bond ':' inside '[]')

set with explicit hydrogens and without recursive environments:
no error or not processable SMARTS

set with explicit hydrogens and recursive environments:
c;H1;$(c([cH])([cH,n&H0,O]))][H] (missing '[]')

Property	max	min	avg	std. dev.	Q(25%)	median	Q(75%)
nodes	49	1	9.23	8.99	2	5	15
aliphatic	38	0	6.17	5.96	2	4	9
aromatic	49	0	7.96	9.73	0	2	15
aliphatic and aromatic	38	0	4.90	6.59	0	1	9
wildcard	8	0	0.31	0.87	0	0	0
aliphaticWildcard	8	0	0.02	0.25	0	0	0
aromaticWildcard	20	0	0.10	0.88	0	0	0
degree	7	0	0.20	0.70	0	0	0
explicit Hs	8	0	0.31	0.88	0	0	0
implicit Hs	0	0	0	0	0	0	0
ring member	23	0	4.23	5.35	0	1	8
ring size	5	0	0.02	0.25	0	0	0
valence	1	0	0	0.06	0	0	0
connectivity	10	0	0.33	0.96	0	0	0
ring connectivity	0	0	0	0	0	0	0
charge	2	0	0.04	0.22	0	0	0
periodic number	38	0	4.56	6.61	0	0	9
chirality	1	0	0	0.04	0	0	0
mass	0	0	0	0	0	0	0
recursion	4	0	0.33	0.65	0	0	0
logicals	5	0	0.32	0.70	0	0	0
edges	50	0	9.03	9.89	1	4	16
single bonds	38	0	5.27	6.09	1	3	9
double bonds	5	0	0.85	1.07	0	0	1
triple bonds	3	0	0.07	0.32	0	0	0
ring bonds	5	0	0.19	0.45	0	0	0
aromatic bonds	23	0	3.43	5.03	0	0	6
any bonds	15	0	0.12	0.79	0	0	0
directional bonds	4	0	0.01	0.16	0	0	0
logicals	23	0	0.73	2.22	0	0	0

Table 1: Profile over the number of property occurrences of all 1235 SMARTS substructures.

Property	max	min	avg	std. dev.	Q(25%)	median	Q(75%)
nodes	20	1	3.39	2.60	2	2	4
aliphatic	17	0	2.66	1.99	1	2	4
aromatic	20	0	1.32	2.19	0	1	2
aliphatic and aromatic	10	0	0.59	1.15	0	0	1
wildcard	8	0	0.38	0.99	0	0	0
aliphaticWildcard	8	0	0.03	0.33	0	0	0
aromaticWildcard	20	0	0.16	1.14	0	0	0
degree	7	0	0.34	0.89	0	0	0
explicit Hs	8	0	0.51	1.10	0	0	1
implicit Hs	0	0	0	0	0	0	0
ring member	20	0	1.07	2.44	0	0	1
ring size	5	0	0.03	0.33	0	0	0
valence	1	0	0.01	0.07	0	0	0
connectivity	10	0	0.46	1.18	0	0	0
ring connectivity	0	0	0	0	0	0	0
charge	2	0	0.05	0.25	0	0	0
periodic number	5	0	0.21	0.61	0	0	0
chirality	1	0	0	0.05	0	0	0
mass	0	0	0	0	0	0	0
recursion	2	0	0.40	0.67	0	0	1
logicals	5	0	0.39	0.74	0	0	1
edges	23	0	2.56	2.98	1	1	3
single bonds	23	0	1.86	2.76	0	1	2
double bonds	4	0	0.43	0.73	0	0	1
triple bonds	1	0	0.02	0.15	0	0	0
ring bonds	4	0	0.29	0.51	0	0	1
aromatic bonds	23	0	0.74	2.28	0	0	0
any bonds	9	0	0.14	0.70	0	0	0
directional bonds	4	0	0.01	0.21	0	0	0
logicals	23	0	0.67	2.25	0	0	0

Table 2: Profile over the number of property occurrences of 738 SMARTS substructures without explicit hydrogens.

Property	max	min	avg	std. dev.	Q(25%)	median	Q(75%)
nodes	49	1	17.92	8.04	12	17	23
aliphatic	38	0	11.39	6.07	7	11	15
aromatic	49	0	17.84	8.08	12	17	23
aliphatic and aromatic	38	0	11.31	6.10	7	11	15
wildcard	5	0	0.21	0.64	0	0	0
aliphaticWildcard	0	0	0	0	0	0	0
aromaticWildcard	0	0	0	0	0	0	0
degree	0	0	0	0	0	0	0
explicit Hs	1	0	0.01	0.10	0	0	0
implicit Hs	0	0	0	0	0	0	0
ring member	23	0	8.92	5.03	6	9	12
ring size	0	0	0	0	0	0	0
valence	0	0	0	0	0	0	0
connectivity	3	0	0.14	0.43	0	0	0
ring connectivity	0	0	0	0	0	0	0
charge	1	0	0.02	0.15	0	0	0
periodic number	38	0	11.02	6.18	7	11	15
chirality	0	0	0	0	0	0	0
mass	0	0	0	0	0	0	0
recursion	4	0	0.21	0.60	0	0	0
logicals	4	0	0.23	0.62	0	0	0
edges	50	0	18.65	8.67	13	18	24
single bonds	38	0	10.34	6.15	6	10	14
double bonds	5	0	1.47	1.19	0	1	2
triple bonds	3	0	0.14	0.46	0	0	0
ring bonds	5	0	0.04	0.29	0	0	0
aromatic bonds	23	0	7.42	5.35	3	6	11
any bonds	15	0	0.10	0.92	0	0	0
directional bonds	0	0	0	0	0	0	0
logicals	10	0	0.82	2.19	0	0	0

Table 3: Profile over the number of property occurrences of 497 SMARTS substructures with explicit hydrogens.

Property	max	min	avg	std. dev.	Q(25%)	median	Q(75%)
nodes	49	1	10.52	9.28	3	7	17
aliphatic	38	0	7.02	6.20	2	5	11
aromatic	49	0	9.11	10.24	0	5	17
aliphatic and aromatic	38	0	5.61	7	0	2	11
wildcard	8	0	0.18	0.84	0	0	0
aliphaticWildcard	8	0	0.02	0.29	0	0	0
aromaticWildcard	20	0	0.12	1.01	0	0	0
degree	7	0	0.16	0.72	0	0	0
explicit Hs	8	0	0.36	0.96	0	0	0
implicit Hs	0	0	0	0	0	0	0
ring member	23	0	4.84	5.62	0	3	9
ring size	5	0	0.03	0.29	0	0	0
valence	1	0	0	0.07	0	0	0
connectivity	10	0	0.34	1.03	0	0	0
ring connectivity	0	0	0	0	0	0	0
charge	2	0	0.05	0.24	0	0	0
periodic number	38	0	5.38	7.07	0	1	11
chirality	1	0	0	0.05	0	0	0
mass	0	0	0	0	0	0	0
recursion	0	0	0	0	0	0	0
logicals	5	0	0.19	0.55	0	0	0
edges	50	0	10.46	10.20	2	7	18
single bonds	38	0	6.06	6.39	1	4	10
double bonds	5	0	1.01	1.10	0	1	2
triple bonds	3	0	0.09	0.35	0	0	0
ring bonds	5	0	0.15	0.44	0	0	0
aromatic bonds	23	0	3.93	5.27	0	0	6
any bonds	15	0	0.16	0.91	0	0	0
directional bonds	4	0	0.01	0.18	0	0	0
logicals	23	0	0.81	2.36	0	0	0

Table 4: Profile over the number of property occurrences of 936 SMARTS substructures without recursive atom environments.

Property	max	min	avg	std. dev.	Q(25%)	median	Q(75%)
nodes	34	1	5.21	6.53	1	2	7
aliphatic	23	0	3.52	4.13	1	2	4
aromatic	34	0	4.40	6.80	1	1	6
aliphatic and aromatic	23	0	2.71	4.40	0	1	2
wildcard	5	0	0.72	0.83	0	1	1
aliphaticWildcard	1	0	0	0.06	0	0	0
aromaticWildcard	1	0	0.01	0.08	0	0	0
degree	2	0	0.33	0.65	0	0	0
explicit Hs	5	0	0.15	0.53	0	0	0
implicit Hs	0	0	0	0	0	0	0
ring member	21	0	2.32	3.78	0	0	2
ring size	0	0	0	0	0	0	0
valence	0	0	0	0	0	0	0
connectivity	5	0	0.30	0.72	0	0	0
ring connectivity	0	0	0	0	0	0	0
charge	1	0	0.02	0.14	0	0	0
periodic number	19	0	1.98	3.92	0	0	2
chirality	0	0	0	0	0	0	0
mass	0	0	0	0	0	0	0
recursion	4	0	1.34	0.62	1	1	2
logicals	4	0	0.76	0.91	0	1	1
edges	37	0	4.59	7.21	0	1	6
single bonds	17	0	2.79	4.15	0	1	4
double bonds	4	0	0.32	0.77	0	0	0
triple bonds	3	0	0.02	0.20	0	0	0
ring bonds	1	0	0.31	0.46	0	0	1
aromatic bonds	22	0	1.88	3.80	0	0	1
any bonds	2	0	0.01	0.14	0	0	0
directional bonds	0	0	0	0	0	0	0
logicals	10	0	0.46	1.72	0	0	0

Table 5: Profile over the number of property occurrences of 299 SMARTS substructures with recursive atom environments.

Property	max	min	avg	std. dev.	Q(25%)	median	Q(75%)
nodes	20	1	3.89	2.74	2	3	5
aliphatic	17	0	3.09	2.13	2	3	4
aromatic	20	0	1.32	2.46	0	0	1
aliphatic and aromatic	10	0	0.52	1.28	0	0	0
wildcard	8	0	0.34	1.12	0	0	0
aliphaticWildcard	8	0	0.04	0.39	0	0	0
aromaticWildcard	20	0	0.23	1.37	0	0	0
degree	7	0	0.30	0.95	0	0	0
explicit Hs	8	0	0.66	1.24	0	0	1
implicit Hs	0	0	0	0	0	0	0
ring member	20	0	1.18	2.72	0	0	0
ring size	5	0	0.05	0.39	0	0	0
valence	1	0	0.01	0.09	0	0	0
connectivity	10	0	0.53	1.32	0	0	0
ring connectivity	0	0	0	0	0	0	0
charge	2	0	0.07	0.29	0	0	0
periodic number	5	0	0.18	0.60	0	0	0
chirality	1	0	0	0.06	0	0	0
mass	0	0	0	0	0	0	0
recursion	0	0	0	0	0	0	0
logicals	5	0	0.32	0.70	0	0	0
edges	23	0	3.09	3.17	1	2	4
single bonds	23	0	2.19	3	0	1	3
double bonds	4	0	0.59	0.80	0	0	1
triple bonds	1	0	0.03	0.18	0	0	0
ring bonds	4	0	0.25	0.51	0	0	0
aromatic bonds	23	0	0.82	2.47	0	0	0
any bonds	9	0	0.20	0.84	0	0	0
directional bonds	4	0	0.02	0.25	0	0	0
logicals	23	0	0.76	2.44	0	0	0

Table 6: Profile over the number of property occurrences of 504 SMARTS substructures without hydrogen atoms and without recursion.

Property	max	min	avg	std. dev.	Q(25%)	median	Q(75%)
nodes	11	1	2.33	1.87	1	2	2
aliphatic	8	0	1.75	1.22	1	1	2
aromatic	7	0	1.32	1.43	1	1	2
aliphatic and aromatic	5	0	0.74	0.78	0	1	1
wildcard	4	0	0.47	0.61	0	0	1
aliphaticWildcard	1	0	0	0.07	0	0	0
aromaticWildcard	1	0	0.01	0.09	0	0	0
degree	2	0	0.42	0.71	0	0	1
explicit Hs	5	0	0.18	0.58	0	0	0
implicit Hs	0	0	0	0	0	0	0
ring member	11	0	0.85	1.67	0	0	1
ring size	0	0	0	0	0	0	0
valence	0	0	0	0	0	0	0
connectivity	5	0	0.33	0.75	0	0	0
ring connectivity	0	0	0	0	0	0	0
charge	1	0	0.02	0.13	0	0	0
periodic number	3	0	0.26	0.65	0	0	0
chirality	0	0	0	0	0	0	0
mass	0	0	0	0	0	0	0
recursion	2	0	1.27	0.55	1	1	2
logicals	4	0	0.54	0.80	0	0	1
edges	12	0	1.41	2.11	0	1	1
single bonds	10	0	1.15	1.98	0	1	1
double bonds	2	0	0.09	0.33	0	0	0
triple bonds	1	0	0	0.07	0	0	0
ring bonds	1	0	0.38	0.49	0	0	1
aromatic bonds	9	0	0.59	1.80	0	0	0
any bonds	1	0	0	0.07	0	0	0
directional bonds	0	0	0	0	0	0	0
logicals	9	0	0.45	1.74	0	0	0

Table 7: Profile over the number of property occurrences of 234 SMARTS substructures without hydrogen atoms and with recursion.

Property	max	min	avg	std. dev.	Q(25%)	median	Q(75%)
nodes	49	1	18.27	8.15	12	17	23
aliphatic	38	0	11.62	6.23	7	11	15
aromatic	49	0	18.19	8.17	12	17	23
aliphatic and aromatic	38	0	11.54	6.24	7	11	15
wildcard	1	0	0	0.07	0	0	0
aliphaticWildcard	0	0	0	0	0	0	0
aromaticWildcard	0	0	0	0	0	0	0
degree	0	0	0	0	0	0	0
explicit Hs	1	0	0	0.07	0	0	0
implicit Hs	0	0	0	0	0	0	0
ring member	23	0	9.11	5.09	6	9	12
ring size	0	0	0	0	0	0	0
valence	0	0	0	0	0	0	0
connectivity	2	0	0.12	0.39	0	0	0
ring connectivity	0	0	0	0	0	0	0
charge	1	0	0.02	0.15	0	0	0
periodic number	38	0	11.45	6.28	7	11	15
chirality	0	0	0	0	0	0	0
mass	0	0	0	0	0	0	0
recursion	0	0	0	0	0	0	0
logicals	2	0	0.03	0.20	0	0	0
edges	50	0	19.05	8.76	13	18	25
single bonds	38	0	10.58	6.33	6	10	14
double bonds	5	0	1.51	1.18	1	1	2
triple bonds	3	0	0.15	0.47	0	0	0
ring bonds	5	0	0.04	0.30	0	0	0
aromatic bonds	23	0	7.56	5.36	4	6	12
any bonds	15	0	0.11	0.98	0	0	0
directional bonds	0	0	0	0	0	0	0
logicals	10	0	0.87	2.26	0	0	0

Table 8: Profile over the number of property occurrences of 432 SMARTS substructures with hydrogen atoms and without recursion.

Property	max	min	avg	std. dev.	Q(25%)	median	Q(75%)
nodes	34	1	15.58	6.77	11	16	20
aliphatic	23	1	9.91	4.58	7	10	13
aromatic	34	0	15.49	6.96	11	16	20
aliphatic and aromatic	23	0	9.82	4.76	7	10	13
wildcard	5	0	1.58	0.94	1	1	2
aliphaticWildcard	0	0	0	0	0	0	0
aromaticWildcard	0	0	0	0	0	0	0
degree	0	0	0	0	0	0	0
explicit Hs	1	0	0.05	0.21	0	0	0
implicit Hs	0	0	0	0	0	0	0
ring member	21	0	7.65	4.40	5	6	11
ring size	0	0	0	0	0	0	0
valence	0	0	0	0	0	0	0
connectivity	3	0	0.22	0.59	0	0	0
ring connectivity	0	0	0	0	0	0	0
charge	1	0	0.03	0.17	0	0	0
periodic number	19	0	8.14	4.53	5	8	11
chirality	0	0	0	0	0	0	0
mass	0	0	0	0	0	0	0
recursion	4	1	1.58	0.78	1	1	2
logicals	4	0	1.52	0.88	1	1	2
edges	37	0	16.03	7.48	11	17	21
single bonds	17	0	8.72	4.47	5	9	12
double bonds	4	0	1.15	1.22	0	1	2
triple bonds	3	0	0.08	0.40	0	0	0
ring bonds	1	0	0.06	0.24	0	0	0
aromatic bonds	22	0	6.54	5.20	1	6	11
any bonds	2	0	0.05	0.27	0	0	0
directional bonds	0	0	0	0	0	0	0
logicals	10	0	0.51	1.64	0	0	0

Table 9: Profile over the number of property occurrences of 65 SMARTS substructures with hydrogen atom and with recursion.

Property	max	min	avg	std. dev.	Q(25%)	up. median	Q(75%)
nodes	22	1	4.48	3.97	2	3	6
aliphatic	20	0	3.35	2.79	2	3	4
aromatic	22	0	2.78	4.47	0	1	4
aliphatic and aromatic	20	0	1.65	3.05	0	0	2
wildcard	8	0	0.45	1.16	0	0	0
aliphaticWildcard	8	0	0.04	0.40	0	0	0
aromaticWildcard	16	0	0.16	0.94	0	0	0
degree	6	0	0.27	0.82	0	0	0
explicit Hs	7	0	0.52	1.11	0	0	1
implicit Hs	0	0	0	0	0	0	0
ring member	17	0	1.60	3.17	0	0	1
ring size	5	0	0.04	0.32	0	0	0
valence	1	0	0	0.07	0	0	0
connectivity	10	0	0.56	1.26	0	0	1
ring connectivity	0	0	0	0	0	0	0
charge	2	0	0.06	0.25	0	0	0
periodic number	20	0	1.16	2.80	0	0	1
chirality	0	0	0	0	0	0	0
mass	0	0	0	0	0	0	0
recursion	2	0	0.18	0.48	0	0	0
logicals	5	0	0.39	0.76	0	0	1
edges	22	0	3.75	4.48	1	2	5
single bonds	19	0	2.31	3.13	0	1	3
double bonds	4	0	0.53	0.80	0	0	1
triple bonds	3	0	0.04	0.26	0	0	0
ring bonds	5	0	0.24	0.55	0	0	0
aromatic bonds	17	0	1.28	2.96	0	0	0
any bonds	9	0	0.21	0.86	0	0	0
directional bonds	4	0	0.02	0.26	0	0	0
logicals	17	0	0.64	2.16	0	0	0

Table 10: Profile over the number of property occurrences of 469 SMARTS substructures used in ZINC lead-like benchmark set.

Property	max	min	avg	std. dev.	Q(25%)	median	Q(75%)
nodes	17	1	3.53	2.33	2	3	4
aliphatic	11	0	2.81	1.82	2	2	4
aromatic	16	0	1.44	2.44	0	0	2
aliphatic and aromatic	10	0	0.72	1.47	0	0	1
wildcard	8	0	0.48	1.30	0	0	0
aliphaticWildcard	8	0	0.05	0.47	0	0	0
aromaticWildcard	16	0	0.22	1.09	0	0	0
degree	6	0	0.31	0.91	0	0	0
explicit Hs	7	0	0.64	1.21	0	0	1
implicit Hs	0	0	0	0	0	0	0
ring member	16	0	0.91	2.39	0	0	0
ring size	5	0	0.05	0.38	0	0	0
valence	1	0	0.01	0.08	0	0	0
connectivity	10	0	0.62	1.36	0	0	1
ring connectivity	0	0	0	0	0	0	0
charge	2	0	0.07	0.27	0	0	0
periodic number	5	0	0.24	0.69	0	0	0
chirality	0	0	0	0	0	0	0
mass	0	0	0	0	0	0	0
recursion	0	0	0	0	0	0	0
logicals	5	0	0.37	0.75	0	0	1
edges	19	0	2.68	2.68	1	2	3
single bonds	19	0	1.83	2.60	0	1	2
double bonds	4	0	0.45	0.67	0	0	1
triple bonds	1	0	0.02	0.15	0	0	0
ring bonds	4	0	0.25	0.54	0	0	0
aromatic bonds	17	0	0.79	2.40	0	0	0
any bonds	9	0	0.27	0.98	0	0	0
directional bonds	4	0	0.02	0.30	0	0	0
logicals	17	0	0.71	2.36	0	0	0

Table 11: Profile over the number of property occurrences of 347 SMARTS substructures with no hydrogen atoms and no recursion in ZINC lead-like benchmark set.

Property	max	min	avg	std. dev.	Q(25%)	median	Q(75%)
nodes	9.00	1.00	2.52	1.93	2.00	2.00	2.00
aliphatic	8.00	0.00	1.96	1.71	1.00	1.00	2.00
aromatic	6.00	0.00	1.29	1.19	1.00	1.00	2.00
aliphatic and aromatic	2.00	0.00	0.73	0.76	0.00	1.00	1.00
wildcard	2.00	0.00	0.50	0.65	0.00	0.00	1.00
aliphaticWildcard	0.00	0.00	0.00	0.00	0.00	0.00	0.00
aromaticWildcard	0.00	0.00	0.00	0.00	0.00	0.00	0.00
degree	2.00	0.00	0.42	0.67	0.00	0.00	1.00
explicit Hs	5.00	0.00	0.35	0.95	0.00	0.00	0.00
implicit Hs	0.00	0.00	0.00	0.00	0.00	0.00	0.00
ring member	6.00	0.00	0.71	1.38	0.00	0.00	1.00
ring size	0.00	0.00	0.00	0.00	0.00	0.00	0.00
valence	0.00	0.00	0.00	0.00	0.00	0.00	0.00
connectivity	5.00	0.00	0.62	1.13	0.00	0.00	1.00
ring connectivity	0.00	0.00	0.00	0.00	0.00	0.00	0.00
charge	0.00	0.00	0.00	0.00	0.00	0.00	0.00
periodic number	2.00	0.00	0.23	0.59	0.00	0.00	0.00
chirality	0.00	0.00	0.00	0.00	0.00	0.00	0.00
mass	0.00	0.00	0.00	0.00	0.00	0.00	0.00
recursion	2.00	0.00	1.31	0.58	1.00	1.00	2.00
logicals	3.00	0.00	0.67	0.94	0.00	0.00	1.00
edges	9.00	0.00	1.58	2.11	1.00	1.00	1.00
single bonds	9.00	0.00	1.31	1.95	0.00	1.00	1.00
double bonds	2.00	0.00	0.19	0.44	0.00	0.00	0.00
triple bonds	1.00	0.00	0.02	0.14	0.00	0.00	0.00
ring bonds	1.00	0.00	0.42	0.48	0.00	0.00	1.00
aromatic bonds	9.00	0.00	0.42	1.62	0.00	0.00	0.00
any bonds	0.00	0.00	0.00	0.00	0.00	0.00	0.00
directional bonds	0.00	0.00	0.00	0.00	0.00	0.00	0.00
logicals	9.00	0.00	0.35	1.61	0.00	0.00	0.00

Table 12: Profile over the number of property occurrences of 48 SMARTS substructures with no hydrogen atoms and recursion in ZINC lead-like benchmark set.

Property	max	min	avg	std. dev.	Q(25%)	median	Q(75%)
nodes	22	1	10.09	5.39	7	10	13
aliphatic	20	1	6.79	4.14	4	7	8
aromatic	22	0	9.98	5.55	7	10	13
aliphatic and aromatic	20	0	6.68	4.28	4	7	8
wildcard	1	0	0.02	0.13	0	0	0
aliphaticWildcard	0	0	0	0	0	0	0
aromaticWildcard	0	0	0	0	0	0	0
degree	0	0	0	0	0	0	0
explicit Hs	1	0	0.04	0.19	0	0	0
implicit Hs	0	0	0	0	0	0	0
ring member	17	0	5.64	4.34	1	6	9
ring size	0	0	0	0	0	0	0
valence	0	0	0	0	0	0	0
connectivity	2	0	0.25	0.54	0	0	0
ring connectivity	0	0	0	0	0	0	0
charge	1	0	0.04	0.19	0	0	0
periodic number	20	0	6.32	4.23	4	6	8
chirality	0	0	0	0	0	0	0
mass	0	0	0	0	0	0	0
recursion	0	0	0	0	0	0	0
logicals	1	0	0.09	0.29	0	0	0
edges	22	0	10.16	6.05	7	11	15
single bonds	19	0	5.21	4.21	3	4	7
double bonds	4	0	1.36	1.19	0	1	2
triple bonds	3	0	0.12	0.50	0	0	0
ring bonds	5	0	0.14	0.69	0	0	0
aromatic bonds	16	0	3.95	3.97	0	5	6
any bonds	3	0	0.09	0.47	0	0	0
directional bonds	0	0	0	0	0	0	0
logicals	5	0	0.57	1.42	0	0	0

Table 13: Profile over the number of property occurrences of 56 SMARTS substructures with hydrogen atoms and no recursion in ZINC lead-like benchmark set.

Property	max	min	avg	std. dev.	Q(25%)	median	Q(75%)
nodes	22.00	1.00	10.56	6.60	2.00	13.00	15.00
aliphatic	16.00	1.00	6.78	4.31	2.00	7.00	10.00
aromatic	22.00	0.00	10.22	7.06	1.00	13.00	15.00
aliphatic and aromatic	16.00	0.00	6.44	4.73	1.00	7.00	10.00
wildcard	2.00	0.00	1.06	0.62	1.00	1.00	1.00
aliphaticWildcard	0.00	0.00	0.00	0.00	0.00	0.00	0.00
aromaticWildcard	0.00	0.00	0.00	0.00	0.00	0.00	0.00
degree	0.00	0.00	0.00	0.00	0.00	0.00	0.00
explicit Hs	1.00	0.00	0.17	0.37	0.00	0.00	0.00
implicit Hs	0.00	0.00	0.00	0.00	0.00	0.00	0.00
ring member	15.00	0.00	4.61	4.11	0.00	6.00	6.00
ring size	0.00	0.00	0.00	0.00	0.00	0.00	0.00
valence	0.00	0.00	0.00	0.00	0.00	0.00	0.00
connectivity	3.00	0.00	0.33	0.82	0.00	0.00	0.00
ring connectivity	0.00	0.00	0.00	0.00	0.00	0.00	0.00
charge	1.00	0.00	0.06	0.23	0.00	0.00	0.00
periodic number	15.00	0.00	5.28	4.46	0.00	5.00	8.00
chirality	0.00	0.00	0.00	0.00	0.00	0.00	0.00
mass	0.00	0.00	0.00	0.00	0.00	0.00	0.00
recursion	2.00	1.00	1.22	0.42	1.00	1.00	1.00
logicals	2.00	0.00	1.00	0.75	0.00	1.00	2.00
edges	22.00	0.00	10.39	7.24	1.00	13.00	15.00
single bonds	15.00	0.00	5.22	4.25	1.00	5.00	8.00
double bonds	2.00	0.00	0.44	0.76	0.00	0.00	1.00
triple bonds	3.00	0.00	0.17	0.69	0.00	0.00	0.00
ring bonds	0.00	0.00	0.00	0.00	0.00	0.00	0.00
aromatic bonds	16.00	0.00	4.72	4.51	0.00	6.00	6.00
any bonds	0.00	0.00	0.00	0.00	0.00	0.00	0.00
directional bonds	0.00	0.00	0.00	0.00	0.00	0.00	0.00
logicals	3.00	0.00	0.17	0.69	0.00	0.00	0.00

Table 14: Profile over the number of property occurrences of 18 SMARTS substructures with hydrogen atoms and recursion in ZINC lead-like benchmark set.

Property	max	min	avg	std. dev.	Q(25%)	up. median	Q(75%)
nodes	27	1	4.87	4.86	2	3	6
aliphatic	19	0	3.53	3.17	2	2	4
aromatic	27	0	3.04	5.34	0	1	3
aliphatic and aromatic	19	0	1.71	3.39	0	0	2
wildcard	8	0	0.47	1.11	0	0	0
aliphaticWildcard	8	0	0.03	0.37	0	0	0
aromaticWildcard	20	0	0.16	1.18	0	0	0
degree	7	0	0.30	0.87	0	0	0
explicit Hs	8	0	0.52	1.15	0	0	1
implicit Hs	0	0	0	0	0	0	0
ring member	20	0	1.82	3.60	0	0	1
ring size	5	0	0.03	0.29	0	0	0
valence	1	0	0	0.06	0	0	0
connectivity	10	0	0.55	1.29	0	0	1
ring connectivity	0	0	0	0	0	0	0
charge	2	0	0.06	0.27	0	0	0
periodic number	19	0	1.22	3.08	0	0	0
chirality	0	0	0	0	0	0	0
mass	0	0	0	0	0	0	0
recursion	4	0	0.35	0.69	0	0	0
logicals	5	0	0.47	0.83	0	0	1
edges	28	0	4.18	5.47	1	2	5
single bonds	23	0	2.62	3.61	0	1	3
double bonds	4	0	0.51	0.81	0	0	1
triple bonds	3	0	0.03	0.22	0	0	0
ring bonds	5	0	0.25	0.53	0	0	0
aromatic bonds	23	0	1.53	3.53	0	0	0
any bonds	9	0	0.16	0.77	0	0	0
directional bonds	4	0	0.01	0.23	0	0	0
logicals	23	0	0.70	2.32	0	0	0

Table 15: Profile over the number of property occurrences of 588 SMARTS substructures used in ZINC everything benchmark set.

Property	max	min	avg	std. dev.	Q(25%)	median	Q(75%)
nodes	20.00	1.00	3.73	2.58	2.00	3.00	5.00
aliphatic	17.00	0.00	2.98	2.03	2.00	3.00	4.00
aromatic	20.00	0.00	1.38	2.56	0.00	0.00	2.00
aliphatic and aromatic	10.00	0.00	0.62	1.39	0.00	0.00	0.00
wildcard	8.00	0.00	0.41	1.22	0.00	0.00	0.00
aliphaticWildcard	8.00	0.00	0.05	0.44	0.00	0.00	0.00
aromaticWildcard	20.00	0.00	0.24	1.42	0.00	0.00	0.00
degree	7.00	0.00	0.32	0.97	0.00	0.00	0.00
explicit Hs	8.00	0.00	0.69	1.30	0.00	0.00	1.00
implicit Hs	0.00	0.00	0.00	0.00	0.00	0.00	0.00
ring member	20.00	0.00	1.01	2.66	0.00	0.00	0.00
ring size	5.00	0.00	0.04	0.35	0.00	0.00	0.00
valence	1.00	0.00	0.01	0.07	0.00	0.00	0.00
connectivity	10.00	0.00	0.63	1.44	0.00	0.00	1.00
ring connectivity	0.00	0.00	0.00	0.00	0.00	0.00	0.00
charge	2.00	0.00	0.07	0.31	0.00	0.00	0.00
periodic number	5.00	0.00	0.21	0.65	0.00	0.00	0.00
chirality	0.00	0.00	0.00	0.00	0.00	0.00	0.00
mass	0.00	0.00	0.00	0.00	0.00	0.00	0.00
recursion	0.00	0.00	0.00	0.00	0.00	0.00	0.00
logicals	5.00	0.00	0.37	0.75	0.00	0.00	1.00
edges	23.00	0.00	2.90	2.99	1.00	2.00	4.00
single bonds	23.00	0.00	2.04	2.92	0.00	1.00	2.00
double bonds	4.00	0.00	0.51	0.73	0.00	0.00	1.00
triple bonds	1.00	0.00	0.03	0.17	0.00	0.00	0.00
ring bonds	4.00	0.00	0.24	0.53	0.00	0.00	0.00
aromatic bonds	23.00	0.00	0.83	2.57	0.00	0.00	0.00
any bonds	9.00	0.00	0.23	0.91	0.00	0.00	0.00
directional bonds	4.00	0.00	0.02	0.28	0.00	0.00	0.00
logicals	23.00	0.00	0.76	2.54	0.00	0.00	0.00

Table 16: Profile over the number of property occurrences of 400 SMARTS substructures with no hydrogen atoms and no recursion in ZINC everything benchmark set.

Property	max	min	avg	std. dev.	Q(25%)	median	Q(75%)
nodes	9	1	2.50	1.92	1	2	2
aliphatic	8	0	1.86	1.43	1	2	2
aromatic	6	0	1.37	1.45	1	1	2
aliphatic and aromatic	5	0	0.73	0.84	0	1	1
wildcard	2	0	0.45	0.60	0	0	1
aliphaticWildcard	0	0	0	0	0	0	0
aromaticWildcard	1	0	0.01	0.10	0	0	0
degree	2	0	0.47	0.74	0	0	1
explicit Hs	5	0	0.27	0.77	0	0	0
implicit Hs	0	0	0	0	0	0	0
ring member	6	0	0.91	1.61	0	0	1
ring size	0	0	0	0	0	0	0
valence	0	0	0	0	0	0	0
connectivity	5	0	0.44	0.93	0	0	1
ring connectivity	0	0	0	0	0	0	0
charge	0	0	0	0	0	0	0
periodic number	3	0	0.27	0.67	0	0	0
chirality	0	0	0	0	0	0	0
mass	0	0	0	0	0	0	0
recursion	2	0	1.32	0.58	1	1	2
logicals	4	0	0.61	0.90	0	0	1
edges	9	0	1.58	2.15	0	1	1
single bonds	9	0	1.32	2.05	0	1	1
double bonds	2	0	0.11	0.35	0	0	0
triple bonds	1	0	0.01	0.10	0	0	0
ring bonds	1	0	0.40	0.48	0	0	1
aromatic bonds	9	0	0.62	1.84	0	0	0
any bonds	0	0	0	0	0	0	0
directional bonds	0	0	0	0	0	0	0
logicals	9	0	0.50	1.79	0	0	0

Table 17: Profile over the number of property occurrences of 106 SMARTS substructures with no hydrogen atoms and recursion in ZINC everything benchmark set.

Property	max	min	avg	std. dev.	Q(25%)	median	Q(75%)
nodes	27	1	13.19	7.02	8	12	18
aliphatic	19	0	8.02	4.78	4	8	12
aromatic	27	0	13.09	7.11	8	12	18
aliphatic and aromatic	19	0	7.93	4.86	4	8	12
wildcard	0	0	0	0	0	0	0
aliphaticWildcard	0	0	0	0	0	0	0
aromaticWildcard	0	0	0	0	0	0	0
degree	0	0	0	0	0	0	0
explicit Hs	0	0	0	0	0	0	0
implicit Hs	0	0	0	0	0	0	0
ring member	18	0	7.23	5.41	0	6	11
ring size	0	0	0	0	0	0	0
valence	0	0	0	0	0	0	0
connectivity	2	0	0.35	0.68	0	0	0
ring connectivity	0	0	0	0	0	0	0
charge	1	0	0.05	0.21	0	0	0
periodic number	19	0	7.74	4.86	4	8	12
chirality	0	0	0	0	0	0	0
mass	0	0	0	0	0	0	0
recursion	0	0	0	0	0	0	0
logicals	1	0	0.05	0.21	0	0	0
edges	28	0	13.60	7.86	8	13	20
single bonds	19	0	6.60	5.03	3	5	10
double bonds	4	0	1.16	1.20	0	1	2
triple bonds	2	0	0.07	0.33	0	0	0
ring bonds	5	0	0.14	0.76	0	0	0
aromatic bonds	21	0	6.35	5.72	0	6	11
any bonds	0	0	0	0	0	0	0
directional bonds	0	0	0	0	0	0	0
logicals	5	0	0.58	1.51	0	0	0

Table 18: Profile over the number of property occurrences of 43 SMARTS substructures with hydrogen atoms and no recursion in ZINC everything benchmark set.

Property	max	min	avg	std. dev.	Q(25%)	median	Q(75%)
nodes	23	1	13.74	6.29	10	14	19
aliphatic	18	1	8.77	4.28	5	9	12
aromatic	23	0	13.59	6.59	10	14	19
aliphatic and aromatic	18	0	8.62	4.55	5	9	12
wildcard	4	0	1.54	0.93	1	1	2
aliphaticWildcard	0	0	0	0	0	0	0
aromaticWildcard	0	0	0	0	0	0	0
degree	0	0	0	0	0	0	0
explicit Hs	1	0	0.08	0.27	0	0	0
implicit Hs	0	0	0	0	0	0	0
ring member	15	0	6.69	4.36	5	6	10
ring size	0	0	0	0	0	0	0
valence	0	0	0	0	0	0	0
connectivity	3	0	0.21	0.61	0	0	0
ring connectivity	0	0	0	0	0	0	0
charge	1	0	0.05	0.22	0	0	0
periodic number	16	0	7	4.28	3	7	10
chirality	0	0	0	0	0	0	0
mass	0	0	0	0	0	0	0
recursion	4	1	1.62	0.84	1	1	2
logicals	4	0	1.51	0.98	1	1	2
edges	25	0	14.03	7.01	10	14	20
single bonds	15	0	7.69	4.22	5	8	11
double bonds	4	0	0.97	1.19	0	0	2
triple bonds	3	0	0.10	0.50	0	0	0
ring bonds	1	0	0.05	0.22	0	0	0
aromatic bonds	16	0	5.90	4.80	0	6	11
any bonds	2	0	0.05	0.32	0	0	0
directional bonds	0	0	0	0	0	0	0
logicals	10	0	0.69	1.94	0	0	0

Table 19: Profile over the number of property occurrences of 39 SMARTS substructures with hydrogen atoms and recursion in ZINC everything benchmark set.

Property	max	min	avg	std. dev.	Q(25%)	up. median	Q(75%)
nodes	19	2	10.44	3.84	8	11	12
aliphatic	13	2	6.5	2.65	5	6	8
aromatic	19	2	10.44	3.84	8	11	12
aliphatic and aromatic	13	2	6.5	2.65	5	6	8
wildcard	2	0	0.5	0.71	0	0	1
aliphaticWildcard	0	0	0	0	0	0	0
aromaticWildcard	0	0	0	0	0	0	0
degree	0	0	0	0	0	0	0
explicit Hs	0	0	0	0	0	0	0
implicit Hs	0	0	0	0	0	0	0
ring member	9	1	5.75	1.95	6	6	6
ring size	0	0	0	0	0	0	0
valence	0	0	0	0	0	0	0
connectivity	3	0	0.56	0.93	0	0	1
ring connectivity	0	0	0	0	0	0	0
charge	0	0	0	0	0	0	0
periodic number	11	2	5.44	2.26	4	5	6
chirality	0	0	0	0	0	0	0
mass	0	0	0	0	0	0	0
recursion	2	0	0.5	0.71	0	0	1
logicals	2	0	0.5	0.71	0	0	1
edges	19	1	10.44	4.15	8	11	13
single bonds	13	0	5.19	2.88	4	5	5
double bonds	4	0	1.25	1.25	0	1	2
triple bonds	0	0	0	0	0	0	0
ring bonds	1	0	0.06	0.24	0	0	0
aromatic bonds	10	0	4.12	3.12	0	6	6
any bonds	3	0	0.19	0.73	0	0	0
directional bonds	0	0	0	0	0	0	0
logicals	3	0	0.31	0.85	0	0	0

Table 20: Profile over the number of property occurrences of 16 PAINS patterns.

Properties	min	max	mean	std. dev.	Q(25%)	up. median	Q(75%)
Charge	-5	4	0.08	0.45	0	0	0
# Non-hydrogen atoms	0	27	21.53	2.63	20	22	24
# H-Acceptors	0	14	3.79	1.41	3	4	5
# H-Donors	0	10	1.29	0.94	1	1	2
# Hetero atoms	0	16	6.10	1.54	5	6	7
# Aromatic atoms	0	27	9.60	4.17	6	11	12
# Halogens	0	9	0.39	0.76	0	0	1
# Inorganic atoms	0	0	0.00	0.00	0	0	0
# N + O	0	16	5.31	1.49	4	5	6
# Non-hydrogen bonds	4	32	22.96	3.12	21	23	25
# Rot. bonds	0	19	4.55	2.37	3	4	6
Max CRTB	0	9	2.41	1.22	2	2	3
# Ringsystems	0	5	2.06	0.64	2	2	2
# Arom. ringsystems	0	5	1.43	0.74	1	1	2
# Rings (rel.cycles)	0	10	2.43	0.81	2	2	3
# Ring prototypes	0	10	2.43	0.80	2	2	3
# Arom. rings	0	6	1.74	0.81	1	2	2
Max atoms in ring	0	21	5.94	0.51	6	6	6
Max atoms in ringsys	0	26	7.12	2.07	6	6	9
# R/S centers	0	10	0.61	0.89	0	0	1
# E/Z bonds	0	6	0.05	0.23	0	0	0
MW	70.13	349.98	307.12	32.60	287.08	313.41	333.38
TPSA	0.00	237.95	68.90	23.01	53.85	67.60	82.91
PLogP	-13.41	9.28	2.00	1.44	1.35	2.31	2.98
Volume	83.77	386.92	271.31	32.68	250.70	274.54	294.93

Table 21: Profile for all 2516375 from ZINC lead-like.

Properties	min	max	mean	std. dev.	Q(25%)	up. median	Q(75%)
Charge	-5	6	0.09	0.47	0	0	0
# Non-hydrogen atoms	2	60	26.42	24.19	23	27	29
# H-Acceptors	0	24	4.40	1.73	3	4	6
# H-Donors	0	18	1.35	1.02	1	1	2
# Hetero atoms	0	32	7.08	2.04	6	7	8
# Aromatic atoms	0	54	12.35	5.14	10	12	16
# Halogens	0	26	0.58	0.95	0	0	1
# Inorganic atoms	0	0	0.00	0.00	0	0	0
# N + O	0	26	5.99	1.89	5	6	7
# Non-hydrogen bonds	1	70	28.43	24.03	25	29	32
# Rot. bonds	0	54	5.58	3.12	3	5	7
Max CRTB	0	49	2.65	1.46	2	2	3
# Ringsystems	0	9	2.48	0.83	2	2	3
# Arom. ringsystems	0	8	1.80	0.87	1	2	2
# Rings (rel. cycles)	0	73	3.01	1.10	2	3	4
# Ring prototypes	0	14	3.01	1.09	2	3	4
# Arom. rings	0	11	2.23	0.99	2	2	3
Max atoms in ring	0	38	5.97	0.58	6	6	6
Max atoms in ringsys	0	57	7.60	2.55	6	6	9
# R/S centers	0	20	0.72	1.08	0	0	1
# E/Z bonds	0	11	0.07	0.27	0	0	0
MW	32.06	910.05	378.97	69.86	337.46	381.49	419.49
TPSA	0.00	427.39	76.46	27.92	58.36	74.60	92.32
PLogP	-21.08	16.64	2.85	1.79	1.94	3.06	3.99
Volume	41.29	910.54	331.88	62.58	294.25	332.96	369.63

Table 22: Profile for all 14059666 form ZINC everything.

Properties	min	max	mean	std. dev.	Q(25%)	up. median	Q(75%)
Charge	-4	3	0.05	0.53	0	0	0
# Non-hydrogen atoms	5	27	20.81	3.22	19	21	23
# H-Acceptors	0	12	3.90	1.57	3	4	5
# H-Donors	0	9	1.28	1.07	0	1	2
# Hetero atoms	0	14	6.11	1.76	5	6	7
# Aromatic atoms	0	24	8.76	4.52	6	10	12
# Halogens	0	8	0.43	0.83	0	0	1
# Inorganic atoms	0	0	0.00	0.00	0	0	0
# N + O	0	14	5.23	1.76	4	5	6
# Non-hydrogen bonds	4	31	22.19	3.82	20	23	25
# Rot. bonds	0	17	4.11	2.53	2	4	6
Max CRTB	0	9	2.27	1.39	1	2	3
# Ringsystems	0	5	1.91	0.71	1	2	2
# Arom. ringsystems	0	4	1.24	0.77	1	1	2
# Rings (rel.cycles)	0	7	2.38	0.92	2	2	3
# Ring prototypes	0	7	2.37	0.91	2	2	3
# Arom. rings	0	5	1.58	0.87	1	2	2
Max atoms in ring	0	21	5.88	1.03	6	6	6
Max atoms in ringsys	0	25	7.36	2.70	6	6	9
# R/S centers	0	10	0.73	1.16	0	0	1
# E/Z bonds	0	6	0.09	0.31	0	0	0
MW	100.15	349.98	300.85	37.62	278.22	308.02	331.21
TPSA	0.00	221.10	69.90	27.86	51.54	67.84	85.88
PLogP	-8.92	8.23	1.97	1.57	1.27	2.30	3.01
Volume	94.49	365.79	260.75	38.62	237.15	265.23	288.71

Table 23: Profile 61500 molecules selected from ZINC lead-like for the substructure search set.

Properties	min	max	mean	std. dev.	Q(25%)	up. median	Q(75%)
Charge	-5	5	0.04	0.61	0	0	0
# Non-hydrogen atoms	4	58	26.20	6.59	22	26	30
# H-Acceptors	0	24	4.67	2.18	3	4	6
# H-Donors	0	15	1.46	1.32	1	1	2
# Hetero atoms	0	30	7.25	2.65	6	7	9
# Aromatic atoms	0	48	11.23	6.09	6	12	16
# Halogens	0	17	0.58	0.98	0	0	1
# Inorganic atoms	0	0	0.00	0.00	0	0	0
# N + O	0	26	6.09	2.49	4	6	7
# Non-hydrogen bonds	3	64	28.20	7.49	24	28	33
# Rot. bonds	0	44	5.63	3.97	3	5	8
Max CRTB	0	31	2.75	1.99	2	2	4
# Ringsystems	0	8	2.30	0.97	2	2	3
# Arom. ringsystems	0	8	1.55	0.98	1	2	2
# Rings (rel. cycles)	0	12	3.01	1.32	2	3	4
# Ring prototypes	0	12	3.00	1.30	2	3	4
# Arom. rings	0	11	2.03	1.15	1	2	3
Max atoms in ring	0	28	5.91	1.32	6	6	6
Max atoms in ringsys	0	46	8.10	3.62	6	6	10
# R/S centers	0	20	1.12	1.77	0	1	1
# E/Z bonds	0	9	0.11	0.37	0	0	0
MW	58.07	829.00	379.86	90.63	326.29	382.22	434.96
TPSA	0.00	427.39	81.40	39.60	55.73	76.21	99.18
PLogP	-18.52	14.56	2.80	2.13	1.68	3.04	4.16
Volume	45.77	787.25	328.63	82.83	279.54	329.99	377.05

Table 24: Profile 76800 molecules selected from ZINC everything for substructure search set.

Properties	min	max	mean	std. dev.	Q(25%)	up. median	Q(75%)
Charge	-4	3	0.01	0.47	0	0	0
# Non-hydrogen atoms	7	26	20.44	2.90	19	21	23
# H-Acceptors	0	10	3.90	1.42	3	4	5
# H-Donors	0	7	1.03	0.84	0	1	2
# Hetero atoms	2	12	5.66	1.62	5	6	7
# Aromatic atoms	0	25	10.25	3.88	6	11	12
# Halogens	0	6	0.45	0.84	0	0	1
# Inorganic atoms	0	0	0.00	0.00	0	0	0
# N + O	0	11	4.82	1.57	4	5	6
# Non-hydrogen bonds	4	32	21.90	3.38	20	22	24
# Rot. bonds	0	17	3.19	2.09	2	3	5
Max CRTB	0	8	1.77	1.08	1	2	2
# Ringsystems	0	5	2.01	0.70	1	2	2
# Arom. ringsystems	0	5	1.46	0.70	1	1	2
# Rings (rel.cycles)	0	7	2.46	0.76	2	2	3
# Ring prototypes	0	7	2.45	0.75	2	2	3
# Arom. rings	0	6	1.84	0.76	1	2	2
Max atoms in ring	0	6	5.98	0.28	6	6	6
Max atoms in ringsys	0	25	7.44	2.25	6	6	9
# R/S centers	0	10	0.44	0.73	0	0	1
# E/Z bonds	0	4	0.05	0.22	0	0	0
MW	115.19	349.96	293.30	35.82	269.26	296.73	322.35
TPSA	0.00	160.52	64.77	23.85	47.81	63.60	79.79
PLogP	-7.24	8.47	2.29	1.30	1.80	2.59	3.12
Volume	101.51	363.90	252.94	34.49	230.71	230.58	27.92

Table 25: Profile first 100000 molecules selected from ZINC lead-like.

SMARTS	sequential	1 core	SF(1)	2 cores	SF(2)	4 cores	SF(4)	6 cores	SF(6)	8 cores	SF(8)
Pains 1 (opt.)	2130.52	2250.52	0.95	1124.04	1.90	571.96	3.72	394.95	5.39	305.85	6.97
Pains 2 (opt.)	2022.28	2126.51	0.95	1064.94	1.90	537.01	3.77	374.15	5.40	285.21	7.09
Pains 3 (opt.)	43654.23	44353.03	0.98	22389.27	1.95	11441.77	3.82	7990.46	5.46	6161.48	7.09
Pains 4 (opt.)	151108.71	152948.60	0.99	77435.43	1.95	39872.85	3.79	27899.44	5.42	21731.92	6.95
Pains 5 (opt.)	1621.01	1739.23	0.93	856.39	1.89	435.37	3.72	300.66	5.39	232.11	6.98
Pains 6 (opt.)	331.44	442.70	0.75	209.85	1.58	107.58	3.09	71.81	4.63	56.45	5.89
Pains 7 (opt.)	20357.16	20796.51	0.98	10526.87	1.93	5413.15	3.76	3806.67	5.35	2938.15	6.93
Pains 8 (opt.)	1802.52	1927.64	0.94	954.02	1.89	485.02	3.72	337.14	5.36	259.83	6.95
Pains 9 (opt.)	2111.37	2233.45	0.95	1103.49	1.91	562.95	3.75	390.20	5.41	302.71	6.98
Pains 10 (opt.)	15195.46	15659.95	0.97	7855.92	1.93	4037.65	3.76	2824.49	5.38	2173.46	6.99
Pains 11 (opt.)	1349.31	1466.60	0.92	719.40	1.88	364.98	3.70	250.99	5.39	195.60	6.91
Pains 12 (opt.)	2034.22	2143.91	0.95	1059.22	1.92	539.77	3.77	373.48	5.45	286.10	7.12
Pains 13 (opt.)	1253.19	1384.08	0.91	677.59	1.85	344.72	3.64	236.03	5.31	181.98	6.89
Pains 14 (opt.)	48449.45	49347.79	0.98	24940.30	1.94	12878.70	3.76	9004.55	5.38	6998.47	6.92
Pains 15 (opt.)	2314.56	2430.26	0.95	1202.15	1.93	612.85	3.78	423.73	5.47	325.61	7.11
Pains 16 (opt.)	42742.47	43524.07	0.98	22029.90	1.94	11285.97	3.79	7907.82	5.41	6115.53	6.99

Table 26: Ullmann search times in seconds for PAINS substructures as SMARTS in optimized formulation against the complete ZINC lead-like. Scaling factors (SFs) represent the speed up in comparison to the sequential time.

SMARTS	sequential	1 core	SF(1)	2 cores	SF(2)	4 cores	SF(4)	6 cores	SF(6)	8 cores	SF(8)
Pains 1 (opt.)	388.75	407.57	0.95	210.5	1.85	100.5	3.87	69.88	5.56	53.27	7.30
Pains 2 (opt.)	145.34	155.07	0.94	83.38	1.74	46.83	3.10	34.23	4.25	25.7	5.66
Pains 3 (opt.)	1231.1	1283	0.96	683.6	1.80	317.4	3.88	227.63	5.41	181.54	6.78
Pains 4 (opt.)	201.04	208.11	0.97	116.8	1.72	64.81	3.10	47.47	4.24	38.12	5.27
Pains 5 (opt.)	261.54	274.04	0.95	144.4	1.81	70.97	3.69	51.01	5.13	40.98	6.38
Pains 6 (opt.)	85.71	92.88	0.92	49.28	1.74	27.25	3.15	20.61	4.16	15.83	5.41
Pains 7 (opt.)	1274.9	1253.1	1.02	760.7	1.68	319	4.00	227.83	5.60	174.61	7.30
Pains 8 (opt.)	126.31	133.07	0.95	87.94	1.44	48.56	2.60	34.1	3.70	26.45	4.78
Pains 9 (opt.)	352.39	376.71	0.94	236.8	1.49	110.3	3.19	78.79	4.47	61.29	5.75
Pains 10 (opt.)	215.68	277.62	0.78	149.3	1.44	73.75	2.92	52.84	4.08	41.79	5.16
Pains 11 (opt.)	70.31	81.19	0.87	48.34	1.45	27.13	2.59	20.03	3.51	15.14	4.64
Pains 12 (opt.)	139.19	176.15	0.79	91.61	1.52	52.15	2.67	37.14	3.75	29.44	4.73
Pains 13 (opt.)	125.09	156.87	0.80	74.16	1.69	47.23	2.65	34.86	3.59	26.39	4.74
Pains 14 (opt.)	349.27	409.72	0.85	196.7	1.78	111.3	3.14	78.87	4.43	61.72	5.66
Pains 15 (opt.)	124.37	157.53	0.79	71.77	1.73	46.41	2.68	33.99	3.66	26.28	4.73
Pains 16 (opt.)	872.43	953.21	0.92	499.7	1.75	283.3	3.08	203.4	4.29	162.87	5.36

Table 27: VF2 search times in seconds for PAINS substructures as SMARTS in optimized formulation against the complete ZINC lead-like. Scaling factors (SFs) represent the speed up in comparison to the sequential time.

	time [s]	matches	speedup		time [s]	matches	speedup
PAINS 1				PAINS 9			
original	2439.36	9208	1.00	original	2870.25	7640	1.00
optimized	2242.34	9208	1.09	optimized	2231.74	7640	1.29
anti-opt.	2236.72	9208	1.09	anti-opt.	2886.21	7640	-1.01
PAINS 2				PAINS 10			
original	2635.30	4910	1.00	original	16401.94	13472	1.00
optimized	2130.00	4910	1.24	optimized	16077.22	13472	1.02
anti-opt.	2625.14	4910	1.00	anti-opt.	16460.39	13472	1.00
PAINS 3				PAINS 11			
original	46847.74	511768	1.00	original	1449.60	5208	1.00
optimized	46135.93	511768	1.02	optimized	1403.20	5208	1.03
anti-opt.	46336.84	511768	1.01	anti-opt.	1918.11	5208	-1.32
PAINS 4				PAINS 12			
original	157139.71	11699	1.00	original	3119.04	9056	1.00
optimized	157027.63	11699	1.00	optimized	2142.41	9056	1.46
anti-opt.	154195.33	11699	1.02	anti-opt.	3077.34	9056	1.01
PAINS 5				PAINS 13			
original	1694.23	7520	1.00	original	1764.14	1240	1.00
optimized	1656.71	7520	1.02	optimized	1317.47	1240	1.34
anti-opt.	1929.55	7520	0.88	anti-opt.	1739.60	1240	1.01
PAINS 6				PAINS 14			
original	351.49	6262	1.00	original	51025.77	27364	1.00
optimized	349.54	6262	1.01	optimized	50912.70	27364	1.00
anti-opt.	355.99	6262	-1.01	anti-opt.	50824.62	27364	1.00
PAINS 7				PAINS 15			
original	21770.03	4528	1.00	original	2476.53	2418	1.00
optimized	21070.12	4528	1.03	optimized	2419.29	2418	1.02
anti-opt.	21556.33	4528	1.01	anti-opt.	3193.58	2418	-.128
PAINS 8				PAINS 16			
original	1927.70	5257	1.00	original	44541.86	465080	1.00
optimized	1901.84	5257	1.01	optimized	44478.86	465080	1.00
anti-opt.	2824.82	5257	-1.47	anti-opt.	46449.25	465080	-1.04

Table 28: Ullmann match times in seconds of the PAINS Substructure Set against the complete ZINC lead-like database. All 16 PAINS are given in the original, an optimized, and an anti-optimized substructure formulation in the SI.

	time [s]	matches	speedup		time [s]	matches	speedup
PAINS 1				PAINS 9			
original	642.64	9208	1.00	original	1724.03	7640	1.00
optimized	322.81	9208	1.99	optimized	360.26	7640	4.79
anti-opt.	527.23	9208	1.22	anti-opt.	1684.77	7640	1.02
PAINS 2				PAINS 10			
original	1684.75	4910	1.00	original	1782.21	13472	1.00
optimized	126.01	4910	13.37	optimized	220.01	13472	8.10
anti-opt.	1677.45	4910	1.00	anti-opt.	1744.45	13472	1.02
PAINS 3				PAINS 11			
original	1810.78	511768	1.00	original	197.91	5208	1.00
optimized	1022.14	511768	1.77	optimized	71.74	5208	2.76
anti-opt.	4538.75	511768	-2.51	anti-opt.	356.61	5208	1.80
PAINS 4				PAINS 12			
original	170.42	11699	1.00	original	1698.42	9056	1.00
optimized	168.56	11699	1.01	optimized	142.28	9056	11.94
anti-opt.	2664.49	11699	-15.64	anti-opt.	1675.40	9056	1.01
PAINS 5				PAINS 13			
original	222.96	7520	1.00	original	360.55	1240	1.00
optimized	219.22	7520	1.02	optimized	127.94	1240	2.82
anti-opt.	678.21	7520	-3.04	anti-opt.	355.80	1240	1.01
PAINS 6				PAINS 14			
original	73.32	6262	1.00	original	1849.35	27364	1.00
optimized	71.34	6262	1.03	optimized	355.87	27364	5.20
anti-opt.	97.01	6262	-1.32	anti-opt.	1834.58	27364	1.01
PAINS 7				PAINS 15			
original	1157.12	4528	1.00	original	140.46	2418	1.00
optimized	1137.82	4528	1.02	optimized	128.28	2418	1.09
anti-opt.	1139.51	4528	1.02	anti-opt.	1649.89	2418	11.75
PAINS 8				PAINS 16			
original	240.86	5257	1.00	original	1492.79	465080	1.00
optimized	128.88	5257	1.87	optimized	912.02	465080	1.64
anti-opt.	1649.64	5257	-6.85	anti-opt.	5158.37	465080	3.46

Table 29: VF2 match times in seconds of the PAINS Substructure Set against the complete ZINC lead-like database. All 16 PAINS are given in the original, an optimized, and an anti-optimized substructure formulation in Table 30.

	SMARTS
PAINS 1	
original	[#6]-1(-[#6](~[!#6&!#1]~[#6]-[!#6&!#1]-[#6]-1=[!#6&!#1])~[!#6&!#1])=[#6;!R]-[#1]
optimized	[#6;!R](-[#1])=[#6]-1(-[#6](~[!#6&!#1]~[#6]-[!#6&!#1]-[#6]-1=[!#6&!#1])~[!#6&!#1])
anti-optimized	[#1]-[#6;!R]=[#6]-1(-[#6](~[!#6&!#1]~[#6]-[!#6&!#1]-[#6]-1=[!#6&!#1])~[!#6&!#1])
PAINS 2	
original	c:1:c:c(:c(:c:c:1)-[#6]=[#7]-[#7])-[#8]-[#1]
optimized	[#8](-[#1])-c:1:c(-[#6]=[#7]-[#7]):c:c:c:c:1
anti-optimized	[#1]-[#8]-c:1:c:c:c:c:c:1-[#6]=[#7]-[#7]
PAINS 3	
original	[#6](-[#1])(-[#1])-[#7](-[#6](-[#1])-[#1])-c:1:c:c(:c(:c(:c:1)-[$([#1]),$([#6](-[#1])-[#1]),$([#8]-[#6](-[#1])(-[#1])-[#6](-[#1])-[#1])])-[#7])-[#1]
optimized	[#7](-[#6](-[#1])-[#1])(-[#6](-[#1])(-[#1]))-c:1:c:c(:c(:c(:c:1)-[$([#1]),$([#6](-[#1])-[#1]),$([#8]-[#6](-[#1])(-[#1])-[#6](-[#1])-[#1])])-[#7])-[#1]
anti-optimized	[$([#1]),$([#6](-[#1])-[#1]),$([#8]-[#6](-[#1])(-[#1])-[#6](-[#1])-[#1])]-c:1:c:c(:c:c(:c:1-[#7])-[#1])-[#7](-[#6](-[#1])(-[#1]))(-[#6](-[#1])-[#1])
PAINS 4	
original	n:1(c(c(c:2:c:1:c:c:c:c:2-[#1])-[#6;X4]-[#1])-[$([#6](-[#1])-[#1]),$([#6]=,:[!#6&!#1]),$([#6](-[#1])-[#7]),$([#6](-[#1])(-[#6](-[#1])-[#1])-[#6](-[#1])(-[#1])-[#7](-[#1])-[#6](-[#1])-[#1])])-[$([#1]),$([#6](-[#1])-[#1])]
optimized	n:1(c(c(c:2:c:1:c:c:c:c:2-[#1])-[#6;X4]-[#1])-[$([#6](-[#1])-[#1]),$([#6]=,:[!#6&!#1]),$([#6](-[#1])-[#7]),$([#6](-[#1])(-[#6](-[#1])-[#1])-[#6](-[#1])(-[#1])-[#7](-[#1])-[#6](-[#1])-[#1])])-[$([#1]),$([#6](-[#1])-[#1])]
anti-optimized	[$([#1]),$([#6](-[#1])-[#1])]-n:1(c(c(c:2:c:1:c:c:c:c:2-[#1])-[#6;X4]-[#1])-[$([#6](-[#1])-[#1]),$([#6]=,:[!#6&!#1]),$([#6](-[#1])-[#7]),$([#6](-[#1])(-[#6](-[#1])-[#1])-[#6](-[#1])(-[#1])-[#7](-[#1])-[#6](-[#1])-[#1])])
PAINS 5	
original	[!#6&!#1]=[#6]-1-[#6]=,:[#6]-[#6](=[!#6&!#1])-[#6]=,:[#6]-1
optimized	[!#6&!#1]=[#6]-1-[#6]=,:[#6]-[#6](=[!#6&!#1])-[#6]=,:[#6]-1
anti-optimized	[#6]-1=,:[#6]-[#6](=[!#6&!#1])-[#6]=,:[#6]-[#6](=[!#6&!#1])-1
PAINS 6	
original	[#7;!R]=[#7]
optimized	[#7;!R]=[#7]
anti-optimized	[#7]=[#7;!R]
PAINS 7	
original	[#6]-[#6](=[!#6&!#1;!R])-[#6](=[!#6&!#1;!R])-[$([#6]),$([#16](=[#8])=[#8])]
optimized	[#6](=[!#6&!#1;!R])(-[#6])-[#6](=[!#6&!#1;!R])-[$([#6]),$([#16](=[#8])=[#8])]
anti-optimized	[#6]-[#6](=[!#6&!#1;!R])-[#6](=[!#6&!#1;!R])-[$([#6]),$([#16](=[#8])=[#8])]
PAINS 8	
original	[#7]-[#6;X4]-c:1:c:c:c:c:c:1-[#8]-[#1]
optimized	[#8](-[#1])-c:1:c:c:c:c:c:1-[#6;X4]-[#7]
anti-optimized	[#1]-[#8]-c:1:c:c:c:c:c:1-[#6;X4]-[#7]
PAINS 9	
original	c:1:c:c(:c:c:c:1-[#7](-[#6;X4])-[#6;X4])-[#6]=[#6]
optimized	[#7](-[#6;X4])(-[#6;X4])c:1:c:c:c(-[#6]=[#6]):c:c:1
anti-optimized	[#6]=[#6]-c:1:c:c:c(-[#7](-[#6;X4])-[#6;X4]):c:c:1
PAINS 10	
original	c:1:c:c(:c:c:c:1-[#8]-[#6;X4])-[#7](-[#6;X4])-[$([#1]),$([#6;X4])]
optimized	[#8](-[#6;X4])-c:1:c:c:c(-[#7](-[#6;X4])-[$([#1]),$([#6;X4])]):c:c:1
anti-optimized	[$([#1]),$([#6;X4])]-[#7](-[#6;X4])-c:1:c:c:c(-[#8]-[#6;X4]):c:c:1
PAINS 11	
original	[#7]-1-[#6](=[#16])-[#16]-[#6](=[#6])-[#6]-1=[#8]
optimized	[#16]=[#6](-[#7]-1)-[#16]-[#6](=[#6])-[#6]-1=[#8]
anti-optimized	[#6]=[#6]-1-[#6](=[#8])-[#7]-[#6](=[#16])-[#16]-1
PAINS 12	
original	c:1(:c:c:c(:c:c:1)-[#6]=[#7]-[#7])-[#8]-[#1]
optimized	[#8](-[#1])c:1:c:c:c(-[#6]=[#7]-[#7]):c:c:1
anti-optimized	[#1]-[#8]-c:1:c:c:c(-[#6]=[#7]-[#7]):c:c:1
PAINS 13	
original	[#6]-1(=[#6])-[#6]=[#7]-[!#6&!#1]-[#6]-1=[#8]
optimized	[#8]=[#6]-1-[!#6&!#1]-[#7]=[#6]-[#6]-1=[#6]
anti-optimized	[#6]=[#6]-1-[#6]=[#7]-[!#6&!#1]-[#6]-1=[#8]
PAINS 14	
original	c:1:c:c(:c:c:c:1-[#7](-[#6;X4])-[#6;X4])-[#6;X4]-[$([#8]-[#1]),$([#6]=[#6]-[#1]),$([#7]-[#6;X4])]
optimized	[#7](-[#6;X4])(-[#6;X4])-c:1:c:c:c(-[#6;X4]-[$([#8]-[#1]),$([#6]=[#6]-[#1]),$([#7]-[#6;X4])]):c:c:1
anti-optimized	[$([#8]-[#1]),$([#6]=[#6]-[#1]),$([#7]-[#6;X4])]-[#6;X4]-c:1:c:c:c(-[#7](-[#6;X4])-[#6;X4]):c:c:1
PAINS 15	
original	[#8]=[#6]-2-[#6](=!@[#7]-[#7])-c:1:c:c:c:c:c:1-[#7]-2
optimized	[#7]-[#7]=!@[#6]-1-[#6](=[#8])-[#7]-c:2:c:c:c:c:c:2-1
anti-optimized	[#6](=[#8])-2-[#6](=!@[#7]-[#7])-c:1:c:c:c:c:c:1-[#7]-2
PAINS 16	
original	[#6](-[#1])-[#7](-[#6](-[#1])-[#1])-c:1:c(:c(:c(:c(:c:1-[#1])-[$([#1]),$([#6](-[#1])-[#1])])-[#6](-[#1])-[$([#1]),$([#6]-[#1])])-[#1])-[#1]
optimized	[#7](-[#6]-[#1])(-[#6](-[#1])-[#1])-c:1:c(:c(:c(:c(:c:1-[#1])-[$([#1]),$([#6](-[#1])-[#1])])-[#6](-[#1])-[$([#1]),$([#6]-[#1])])-[#1])-[#1]
anti-optimized	[$([#1]),$([#6]-[#1])]-[#6](-[#1])c:1:c(-[$([#1]),$([#6](-[#1])-[#1])]):c(-[#1]):c(-[#7]([#6]-[#1])[#6](-[#1])-[#1]):c(-[#1]):c(-[#1]):1

Table 30: SMARTS expressions used in optimization experiment given as taken from literature (original), optimized by the given rule set (optimized) and anti-optimized applying the rule set in reverse (anti-optimized).
