# Supplementary material for: Systematic benchmark of substructure search in molecular graphs - From Ullmann to VF2
Source: J Cheminform. 2012 Jul 31;4:13. doi: 10.1186/1758-2946-4-13 (PMC3586954; doi:10.1186/1758-2946-4-13)
Supplement: Additional file 2 — Supplementary Information (Additional file2). Table S1. Profile over the number of property occurrences of all 1235 SMARTS sub-structures. Table S2. Profile over the number of property occurrences of 738 SMARTS substructures without explicit hydrogens. Table S3. Profile over the number of property occurrences of 497 SMARTS substructures with explicit hydrogens. Table S4. Profile over the number of property occurrences of 936 SMARTS substructures without recursive atom environments. Table S5. Profile over the number of property occurrences of 299 SMARTS substructures with recursive atom environments. Table S6. Profile over the number of property occurrences of 504 SMARTS substructures without hydrogen atoms and without recursion. Table S7. Profile over the number of property occurrences of 234 SMARTS substructures without hydrogen atoms and with recursion. Table S8. Profile over the number of property occurrences of 432 SMARTS substructures with hydrogen atoms and without recursion. Table S9. Profile over the number of property occurrences of 65 SMARTS substructures with hydrogen atom and with recursion. Table S10. Profile over the number of property occurrences of 469 SMARTS substructures used in ZINC lead-like benchmark set. Table S11. Profile over the number of property occurrences of 347 SMARTS substructures with no hydrogen atoms and no recursion in ZINC lead-like benchmark set. Table S12. Profile over the number of property occurrences of 48 SMARTS substructures with no hydrogen atoms and recursion in ZINC lead-like benchmark set. Table S13. Profile over the number of property occurrences of 56 SMARTS substructures with hydrogen atoms and no recursion in ZINC lead-like benchmark set. Table S14. Profile over the number of property occurrences of 18 SMARTS substructures with hydrogen atoms and recursion in ZINC lead-like benchmark set. Table S15. Profile over the number of property occurrences of 588 SMARTS substructures used in ZINC everything benchmark set. Tab [file 1758-2946-4-13-S2.pdf]

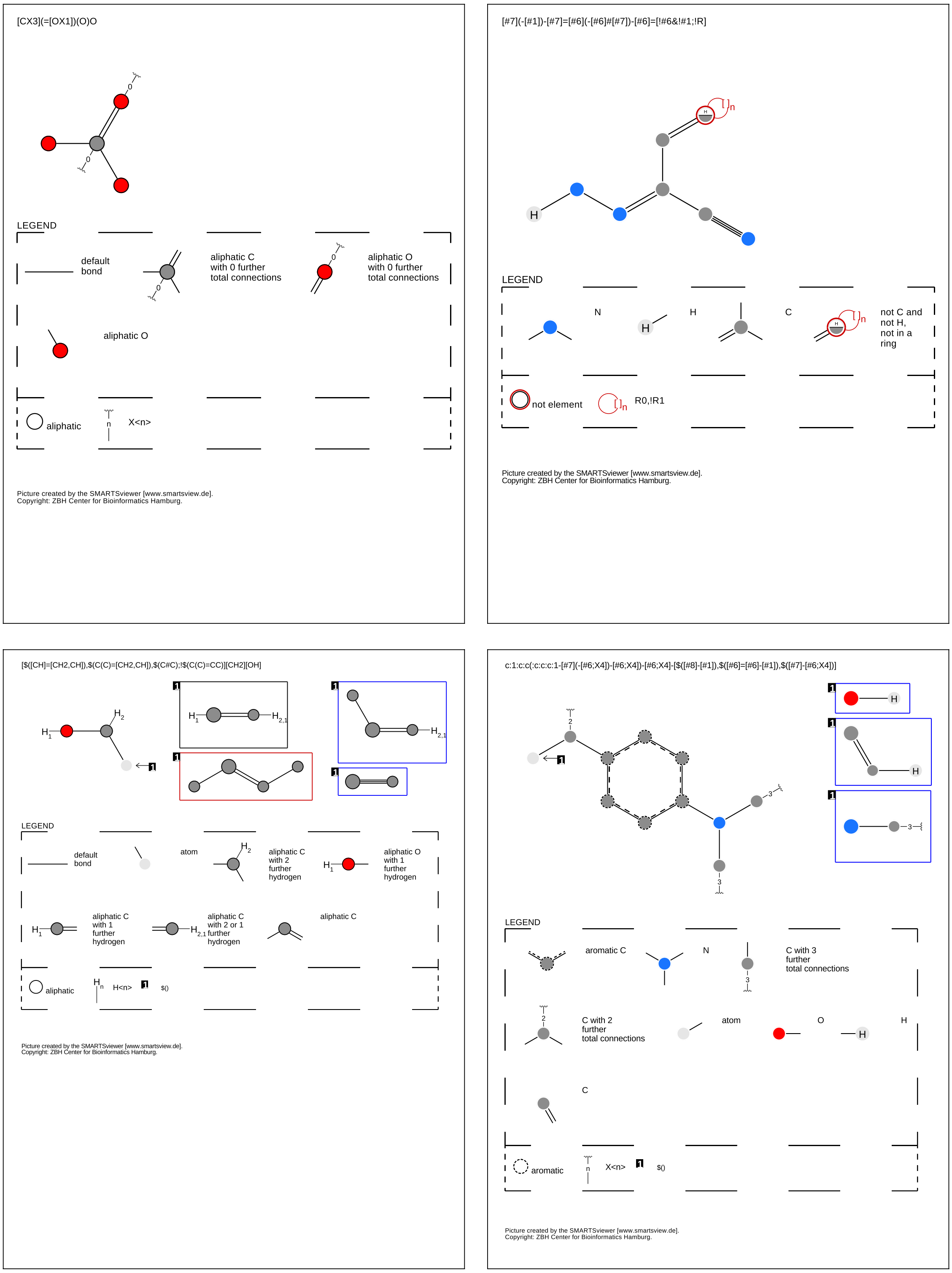

Figure 1: Depiction of SMARTS pattern with no explicit hydrogens and no recursion (top-left), explicit hydrogens and no recursion (top-right), no explicit hydrogens and recursion (bottom-left), and with explicit hydrogens and recursive atom environments (bottom-right). Depictions are created by SMARTSViewer [39].

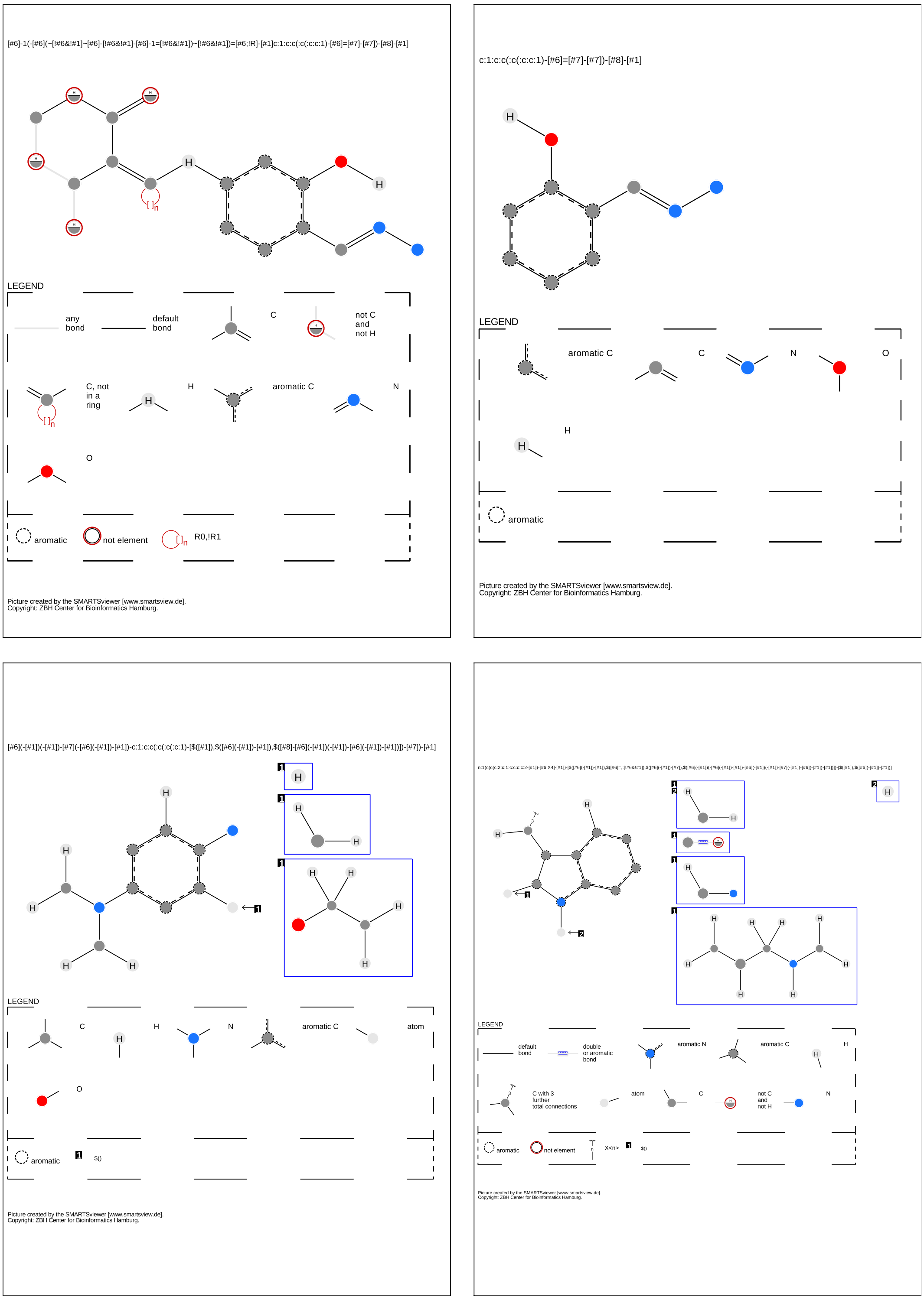

Figure 2: Visual dipiction of PAINS patterns 1-4 created with SMARTSViewer [39].

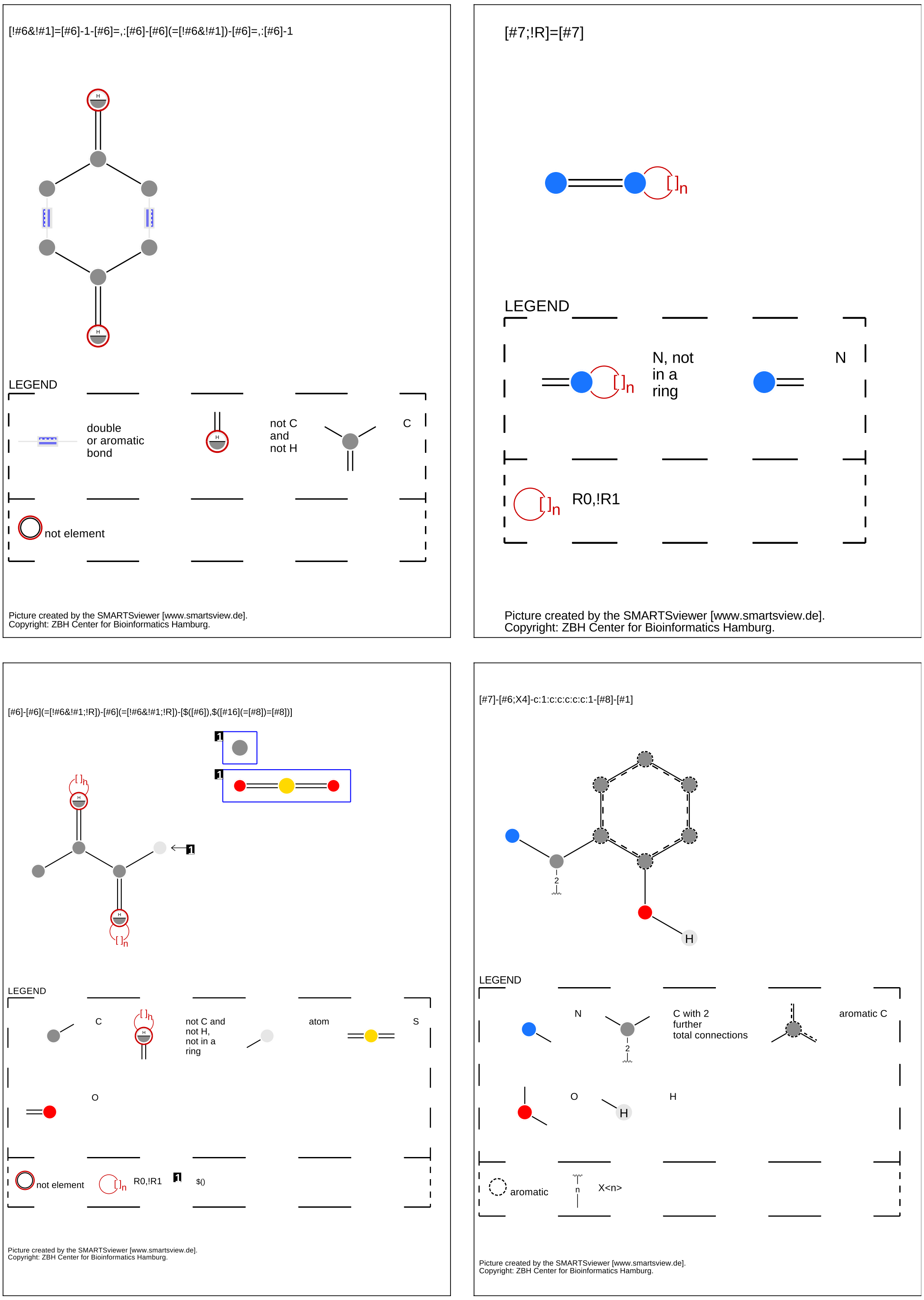

Figure 3: Visual dipiction of PAINS patterns 5-8 created with SMARTSViewer [39].

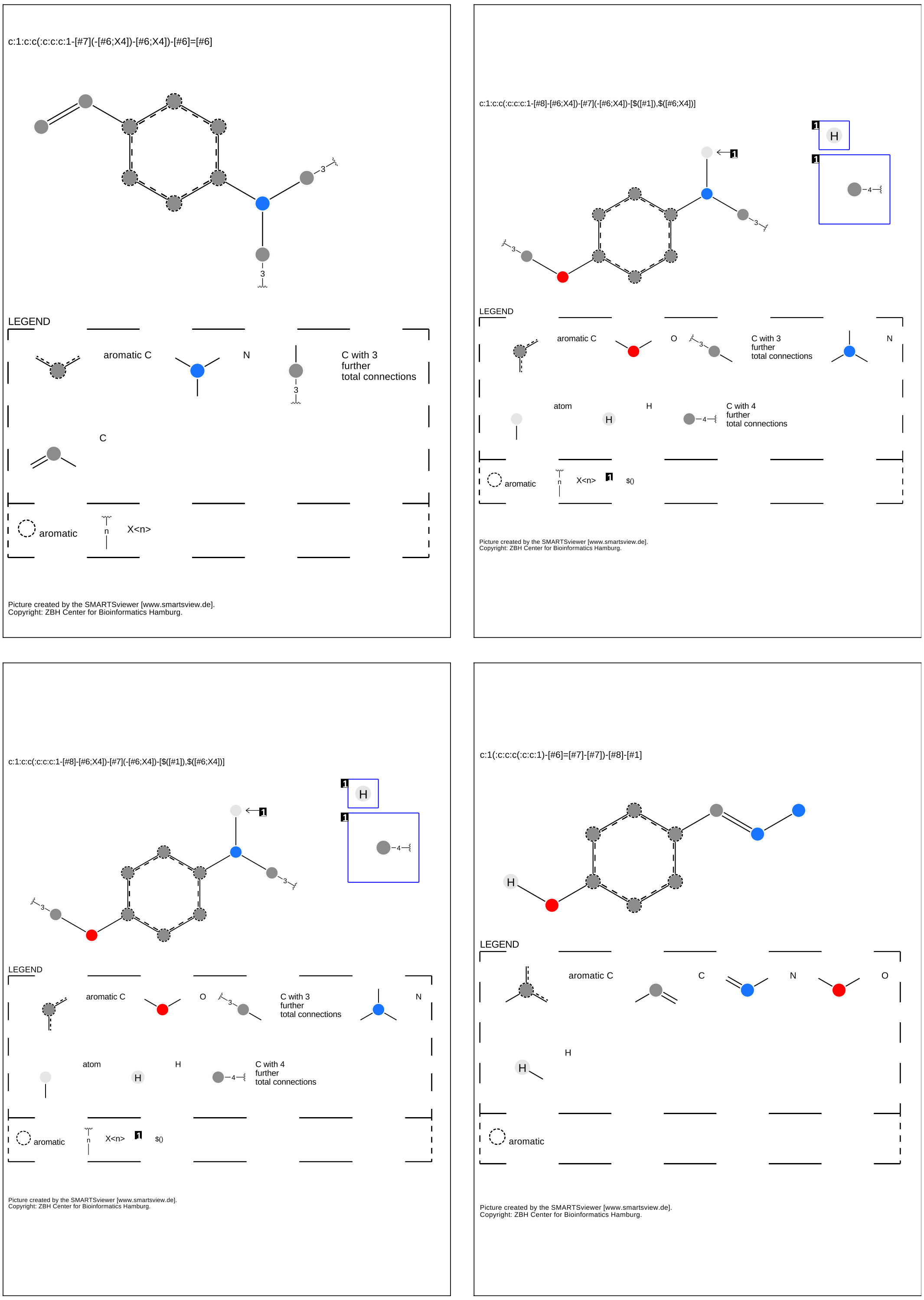

Figure 4: Visual dipiction of PAINS patterns 9-12 created with SMARTSViewer [39].

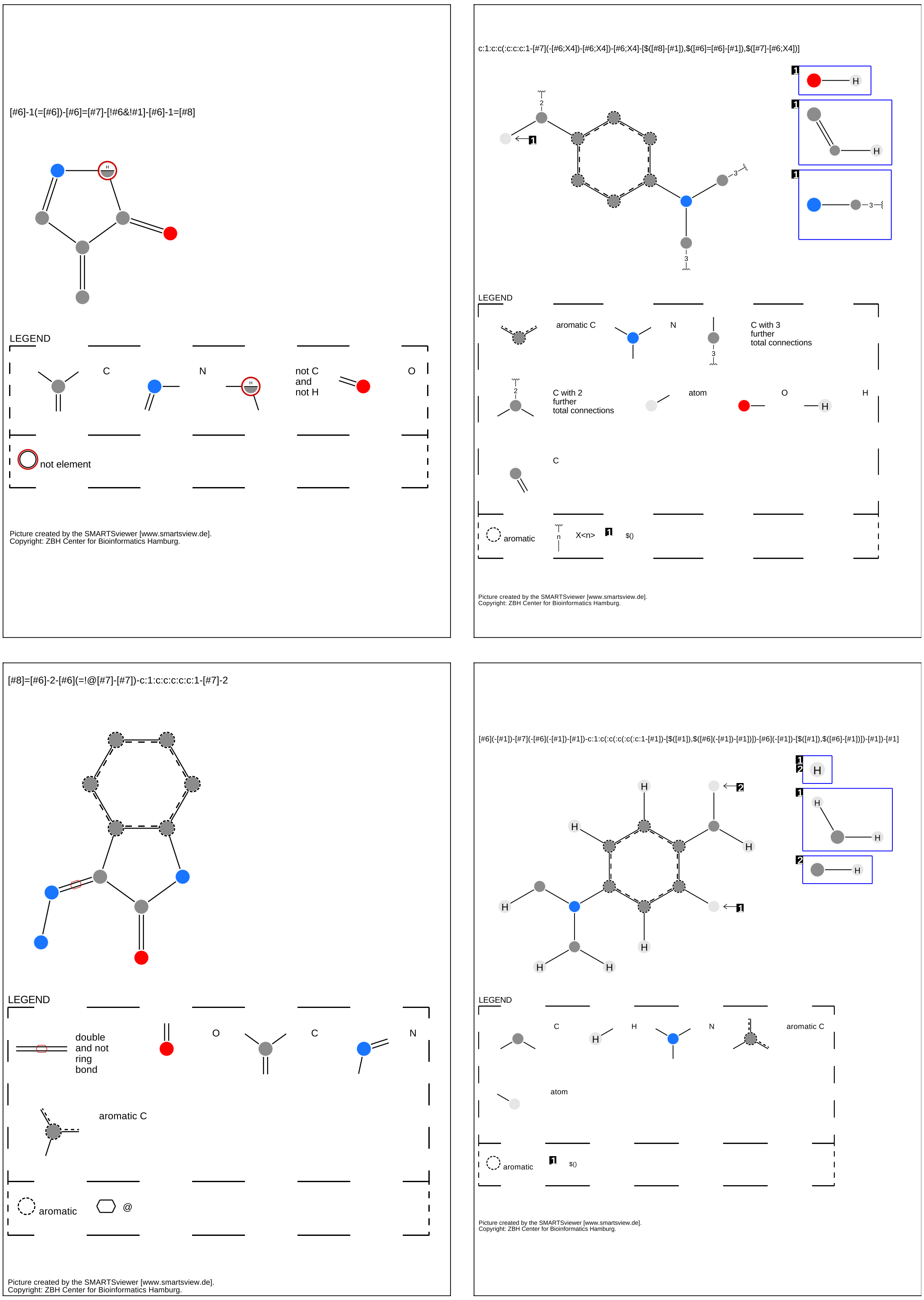

Figure 5: Visual dipiction of PAINS patterns 13-16 created with SMARTSViewer [39].
